# State-dependent intrinsic predictability of cortical network dynamics

**DOI:** 10.1371/journal.pone.0173658

**Published:** 2017-05-04

**Authors:** Leila Fakhraei, Shree Hari Gautam, Woodrow L. Shew

**Affiliations:** Department of Physics, University of Arkansas, Fayetteville, Arkansas, United States of America; McGill University Department of Physiology, CANADA

## Abstract

The information encoded in cortical circuit dynamics is fleeting, changing from moment to moment as new input arrives and ongoing intracortical interactions progress. A combination of deterministic and stochastic biophysical mechanisms governs how cortical dynamics at one moment evolve from cortical dynamics in recently preceding moments. Such temporal continuity of cortical dynamics is fundamental to many aspects of cortex function but is not well understood. Here we study temporal continuity by attempting to predict cortical population dynamics (multisite local field potential) based on its own recent history in somatosensory cortex of anesthetized rats and in a computational network-level model. We found that the intrinsic predictability of cortical dynamics was dependent on multiple factors including cortical state, synaptic inhibition, and how far into the future the prediction extends. By pharmacologically tuning synaptic inhibition, we obtained a continuum of cortical states with asynchronous population activity at one extreme and stronger, spatially extended synchrony at the other extreme. Intermediate between these extremes we observed evidence for a special regime of population dynamics called criticality. Predictability of the near future (10–100 ms) increased as the cortical state was tuned from asynchronous to synchronous. Predictability of the more distant future (>1 s) was generally poor, but, surprisingly, was higher for asynchronous states compared to synchronous states. These experimental results were confirmed in a computational network model of spiking excitatory and inhibitory neurons. Our findings demonstrate that determinism and predictability of network dynamics depend on cortical state and the time-scale of the dynamics.

## Introduction

Concepts like “train of thought” or “stream of consciousness” evoke a picture of ongoing brain function in which thoughts at one moment are inextricably linked with those of the recent past. The neural underpinnings of such temporal continuity of brain activity are largely unknown. At a basic physiological level, it is clear that the action potentials at one moment are caused, in part, by those occurring in the recent past, and those in turn, from earlier neural activity. However, the synaptic interactions that mediate such temporal evolution of neural activity can be strongly modulated, resulting in qualitatively diverse states of neural dynamics, depending on behavioral or pharmacological factors [1,2]. For instance, changes in levels of arousal [3–6], body motility [3], sleep [7], anesthesia [6,8–10], and the balance of excitation and inhibition [11] can incur dramatic changes in dynamics in cerebral cortex. In this context, it stands to reason that, the temporal continuity of neural activity should depend on the cortical state. To test this hypothesis, we measured how well cortical population activity can be predicted based on its own recent history. We interpret the degree of predictability as a quantitative proxy for the degree of temporal continuity. We experimentally measured ongoing population neural activity in the cortex (multi-site local field potential) and in a computational model network of spiking excitatory and inhibitory neurons. We measured predictability across a continuum of different cortical states incurred, in part, by tuning synaptic inhibition. The continuum of states ranged from asynchronous, weakly correlated activity to strongly fluctuating, synchronous activity. Confirmed by our experiments and our model, we found that predictability does indeed exhibit a complex dependence on cortical state. For short term predictions (<50 ms) asynchronous cortical states were less predictable than synchronous states. Surprisingly, the reverse was true for longer term predictions (> 1s)–the synchronous state was less predictable than the asynchronous state. However, all long term predictions were rather poor. Together, our experimental and computational results suggest that temporal continuity of ongoing cortical activity can be dramatically altered by tuning inhibitory synaptic interactions or by other means of tuning the cortical state.

## Results

**Fig 1:**
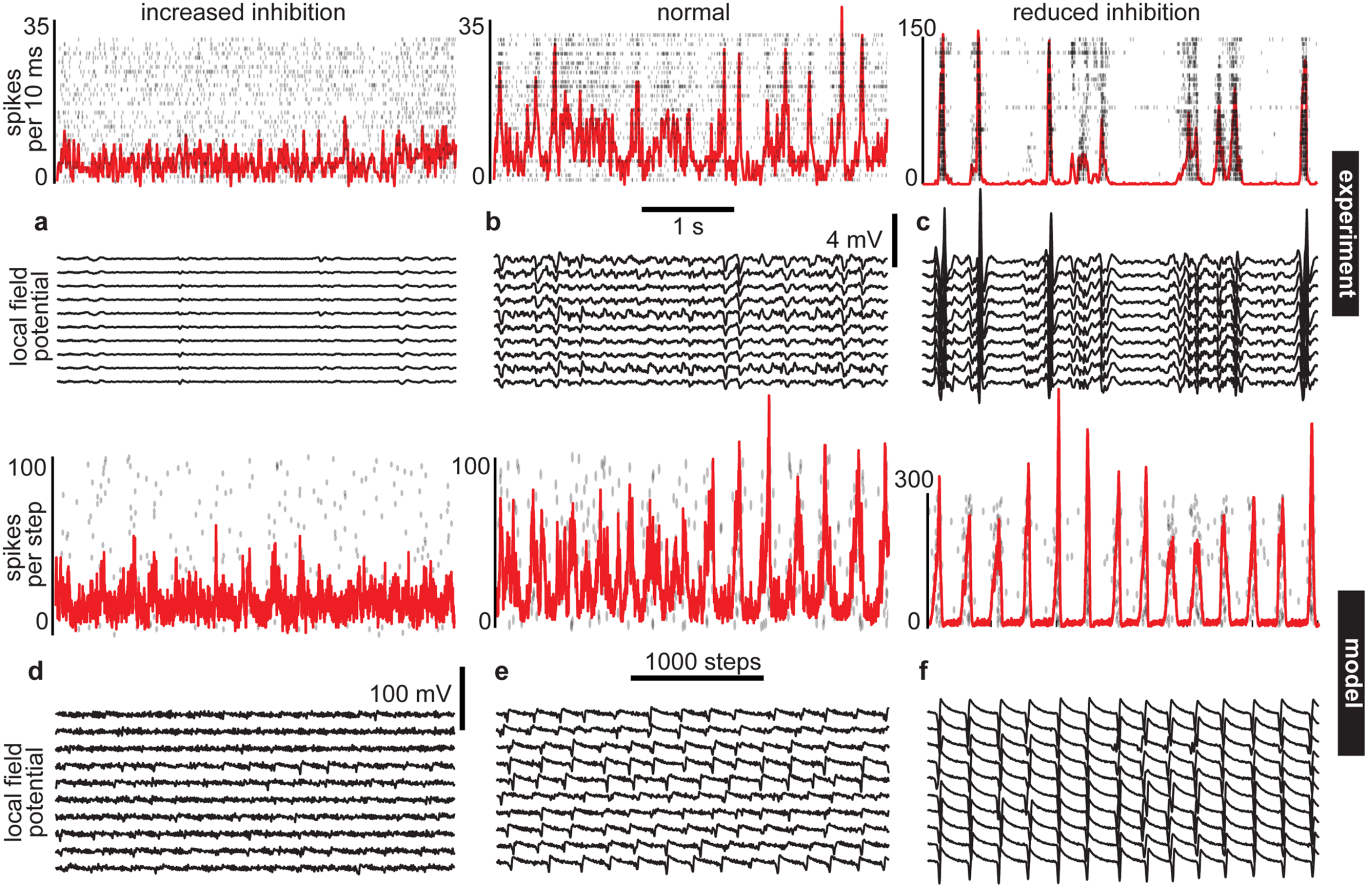
Tuning inhibition to alter the cortical state. For both our experiment (a-c) and our model (d-f), we studied a range of cortical states characterized at one extreme by asynchronous firing and low amplitude LFP (a, d) and at the other extreme by firing synchrony and large amplitude LFP (c, f). These extremes were typically observed when inhibition was increased or decreased, respectively. In between the extremes, population spiking was more varied and LFP was moderate in amplitude (b,e). The shown model examples were computed with *IC* = -75 (increased inhibition), *IC* = -28.5 (normal), and *IC* = -7.5 (reduced inhibition).

We studied temporal continuity and predictability of neuronal network dynamics in experiments and in a computational model. In experiments, we recorded multi-unit activity (MUA) and local field potential (LFP) in somatosensory cortex of anesthetized male rats using 32-channel micro-electrode arrays (Fig 1). Our model consisted of 2952 spiking neurons– 80% excitatory, 20% inhibitory—placed on a two-dimensional grid with spatially localized connectivity. The model neuron dynamics were simulated using established computationally efficient methods [12]. Model spiking activity was compared with experiment MUA. Experimental measurements of LFP were compared with the average membrane potential of groups of model neurons (32 groups, akin to the 32 electrodes in experiments, each group was comprised of 81 neurons in a 9 x 9 grid). In the following results, we will first describe how we imposed changes in cortical state and how we quantitatively assessed such changes. Second, we will describe how we measure predictability and how predictability depends on cortical state.

In both the experiments and the model, we manipulated inhibitory synapses to change the dynamical state of the neuronal population. In experiments, inhibition was manipulated pharmacologically with GABA_A_ agonist muscimol or antagonist bicuculline. Moreover, in experiments, the dynamical state exhibited changes without direct experimental control, as observed in previous studies of urethane anesthetized animals [13,14]. Our data analysis accounts for both the spontaneously occurring shifts in state and the pharmacologically induced shifts, as we describe further below. In both the experiment and the model, we found that enhanced inhibition resulted in asynchronous, low rate population activity, while reduced inhibition typically resulted in large bursts of correlated activity (Fig 1).

**Fig 2:**
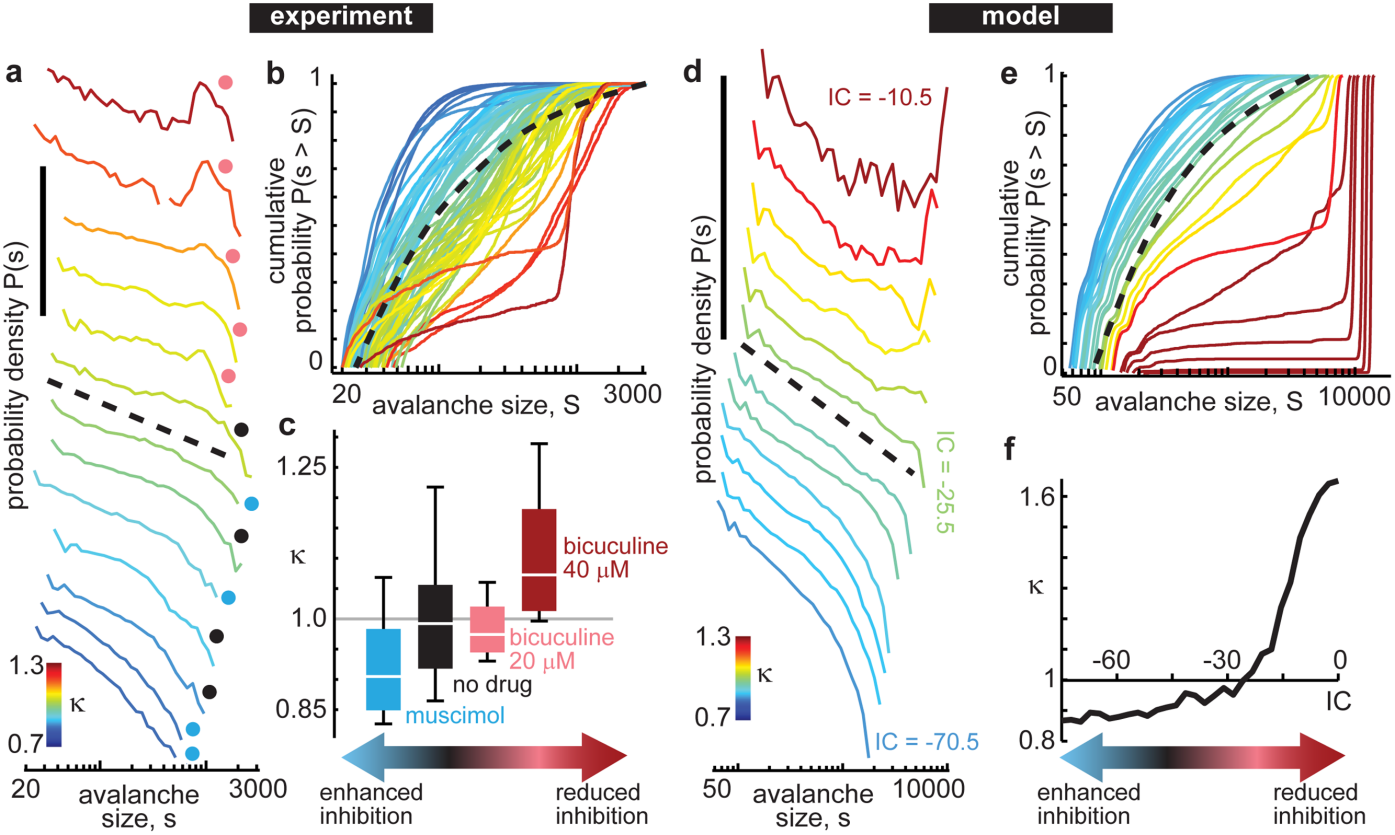
Parameterizing the cortical state based on avalanche distributions. For both the experiment (a-c) and the model (d-f), we indexed the continuum of observed cortical states based on the prevalence of different spatiotemporal scales of activity. **a)** Each line shows one probability density distribution of avalanches obtained from one 20 min experimental recording of ongoing activity (shifted vertically to facilitate comparison of the distribution shapes). Both vertical and horizontal axes are logarithmic. Vertical scale bar indicates 5 orders of magnitude. The colored dot beside each distribution indicates the experimental drug condition (black—no-drug; blue—muscimol; pink—bicuculline 20 μM). A subset of all experiments is shown. Line color indicates the κ value, which measures deviation from a power-law distribution with exponent -1.5 (black dashed line). When large bursts of population activity are dominant κ>1 and when large bursts are absent κ<1. **b)** The probability distributions shown in panel (a) are shown here as cumulative distributions. Color indicates κ, which is defined as the mean of 10 differences (equally spaced across the horizontal axis) between the measured distributions and the reference power-law distribution (black dashed). One distribution for each experiment is shown. **c)** Shown are the upper and lower quartiles, median, and range (box bottom, top, midline, and error bars, respectively) of κ values for each drug condition. Note that decreasing or increasing inhibition systematically increases or decreases κ, respectively. **d-f)** The model results closely parallel the experimental findings. In the model, we treat the pharmacological changes by directly tuning the strength of inhibitory connections (*IC*). Tuning *IC* in the model resulted in a family of avalanche size distributions similar to those observed in the experiments.

We quantitatively assessed changes in the dynamical state of the network based on the prevalence of different spatiotemporal scales of population activity. More specifically, we analyzed distributions of ‘avalanche’ sizes. An avalanche is a period of elevated population activity, which we defined based on MUA spike count time series for the entire population, as other in recent studies [15–17]. In brief, an avalanche is defined as an excursion above a threshold level of spiking. The size of an avalanche is defined as the total number of spikes that occur during this period of above-threshold activity. Distributions of avalanche sizes reveal how often large avalanches occur relative to small avalanches, thus assessing the prevalence of different spatiotemporal scales of population activity (Fig 2a and 2d).

A convenient parameter to index the continuum of observed cortical states, called κ, was developed in previous studies of neuronal avalanches [15,18,19]. Based on cumulative probability distributions (Fig 2b and 2e) κ quantifies how an observed avalanche size distribution deviates from a -1.5 power law. When κ was less than 1, large avalanches were rare and the size distribution was close to exponential in form. When κ was greater than 1, large avalanches were dominant, typically exhibiting a bimodal distribution of sizes. Intermediate between these extremes, avalanches occurred with diverse sizes and the size distribution had a form close to a power law with exponent -1.5. By definition, a perfect match to -1.5 power law corresponds to κ = 1. Importantly, κ allows quantitative comparison of experimental and model results, because κ is readily obtained from both.

**Fig 3:**
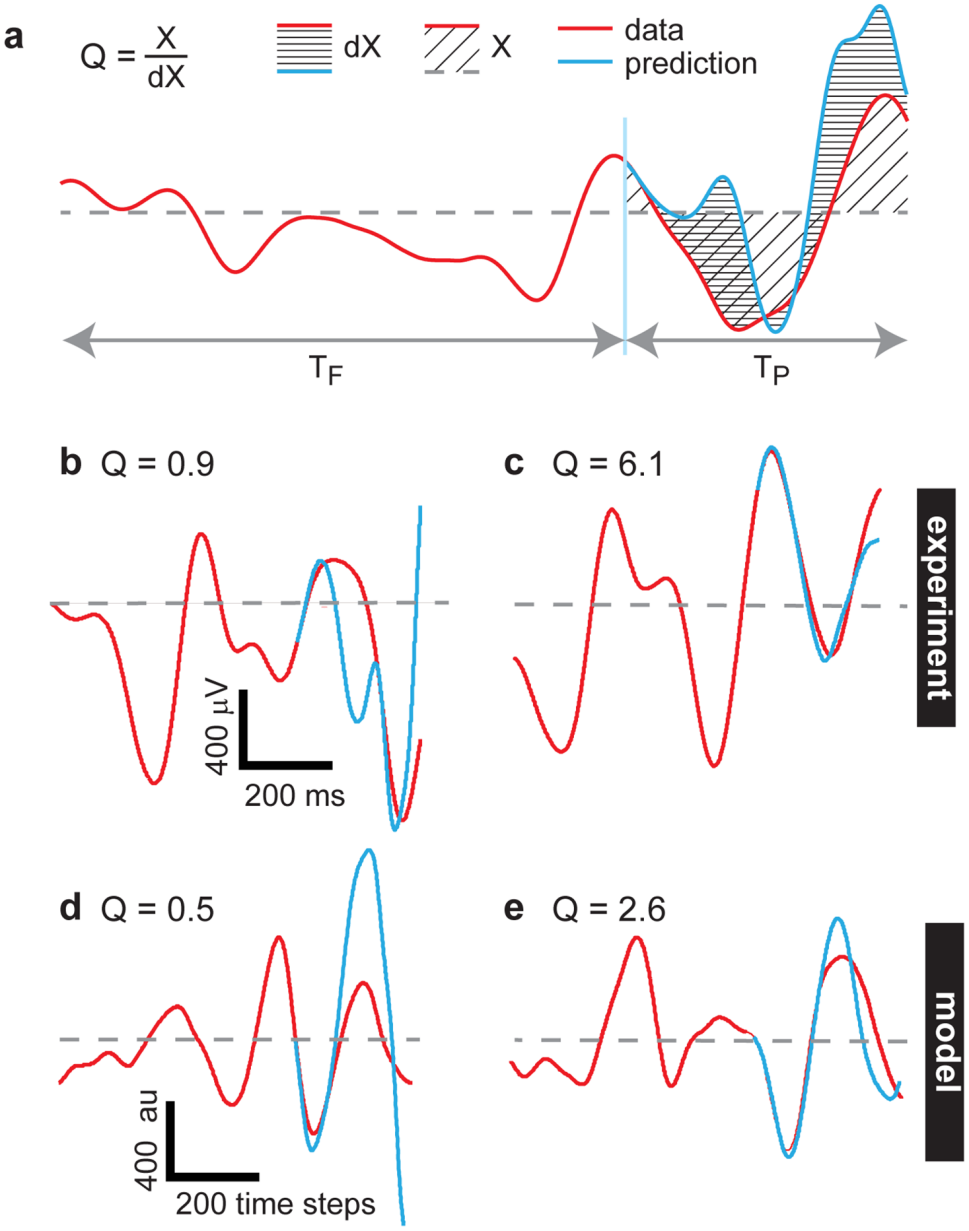
Measuring predictability of multisite LFP. **a)** We fit an autoregressive model to a period of recorded data of duration T_f_. This fit is based on all 32 channels, even though only one channel is shown here. We predict a period of duration T_p_ following the fitting time window. The prediction (blue) is compared with the true measured data (red) to assess the efficacy of the prediction Q. Shown are examples of a poor prediction from the experiment (b), a good prediction from the experiment (c), a poor prediction from the model (d) and a good prediction from the model (e).

**Fig 4:**
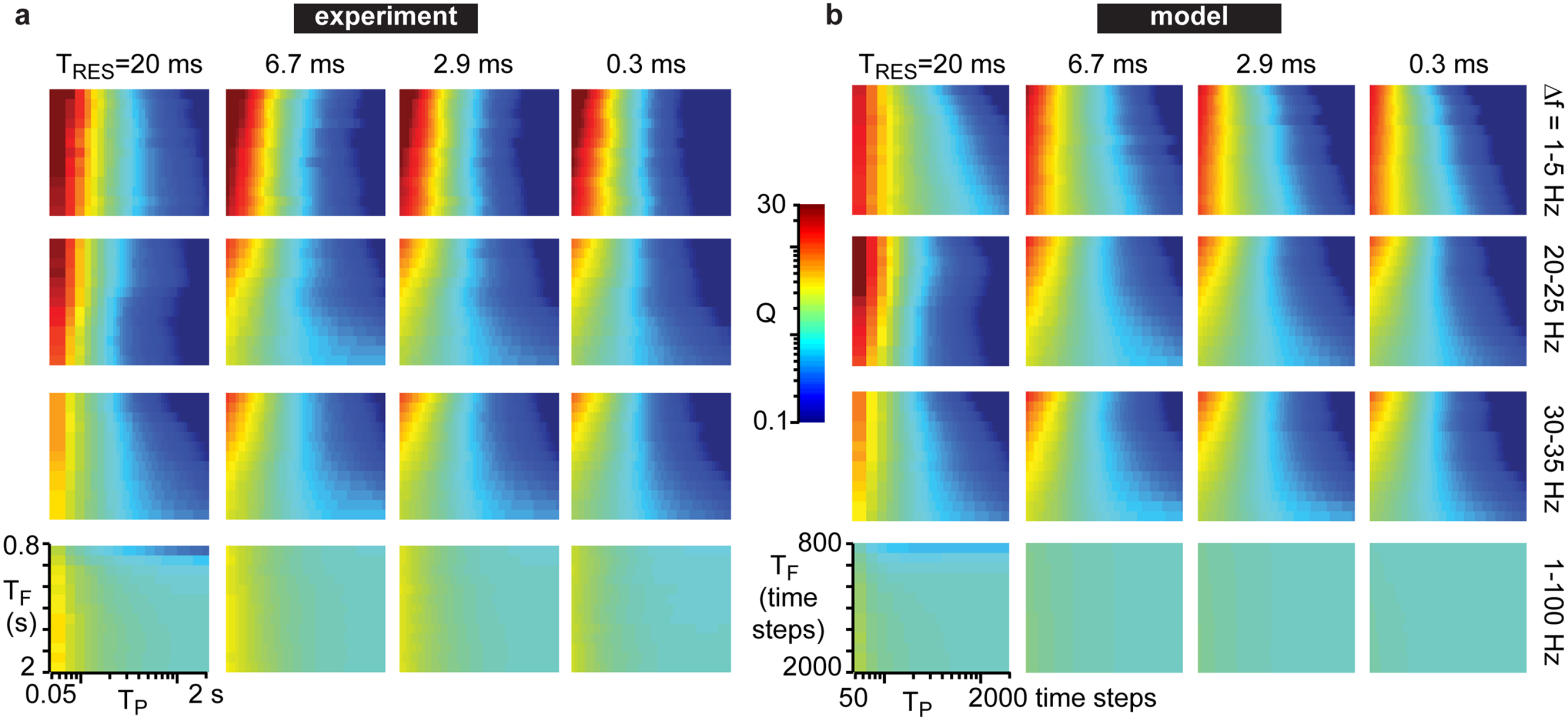
Predictability depends on time scales. Here we show how predictability Q (color) depends on the fitting time T_f_, the prediction time T_p_, time resolution of the recording T_RES_, and frequency band of filtering. The example experiment (a) is from a no-drug recording with κ = 0.99. The example model dataset (b) was simulated with IC = -30 and κ = 0.95. We find good agreement between the experiment and model. Predictions are more sensitive to changes in T_P_ than T_F_. Low frequencies are more predictable than high frequencies for small T_P_, while the opposite is true for longer T_P_.

To determine how the temporal continuity of cortical population dynamics depends on cortical state changes, i.e. changes in κ, we next computed the predictability of population dynamics. We computed predictions of many short periods of ongoing activity and averaged them to assess the overall predictability Q of a given recording. The first step in computing a prediction was to fit an autoregressive model to a short duration T_F_ of local field potential (LFP) recorded from 32 electrodes (Fig 3a), similar to other recent studies [9,20]. After fitting the autoregressive model, it was used to predict LFP during a short time window T_P_ immediately following the fitting time window (Fig 3a). An autoregressive model is meant to handle continuous variables, and thus, is a natural choice for assessing predictability of LFP. Although an autoregressive model is a linear model, and cortical dynamics are certainly nonlinear, it is expected that, over sufficiently short times, nonlinear dynamics can be well approximated by a linear model. Indeed, our predictions were often quite high in quality for short time periods (Figs 3c,3e and 4). However, we do not know *a priori* what duration is sufficiently short for such a linear approximation to be accurate. Indeed, as we will show below, the efficacy of such linear modeling can depend sensitively on changes in cortical state. Rather than pick a single time period T_P_, we studied a range of time windows T_P_ from 50 ms to 2 s. We also examined a range of fitting time window durations T_F_ from 0.8 to 2 s. As expected, shorter times were much more predictable than longer times, i.e. prediction quality Q was highest for short T_P_; Q also depended on T_F_, but not as strongly as it depended on T_P_ (Fig 4). We also note that the prediction is more effective in restricted frequency bands, compared to broadband LFP (Fig 4), generally slow frequencies (1–5 Hz) were most accurately predicted. Moreover, the time resolution of the data (i.e. the sample rate) can also significantly influence predictability (Fig 4). Generally, we found that predictability depended on T_P_, T_F_, frequency band, and time resolution in a similar way for the experiment (Fig 4a) and the model (Fig 4b).

**Fig 5:**
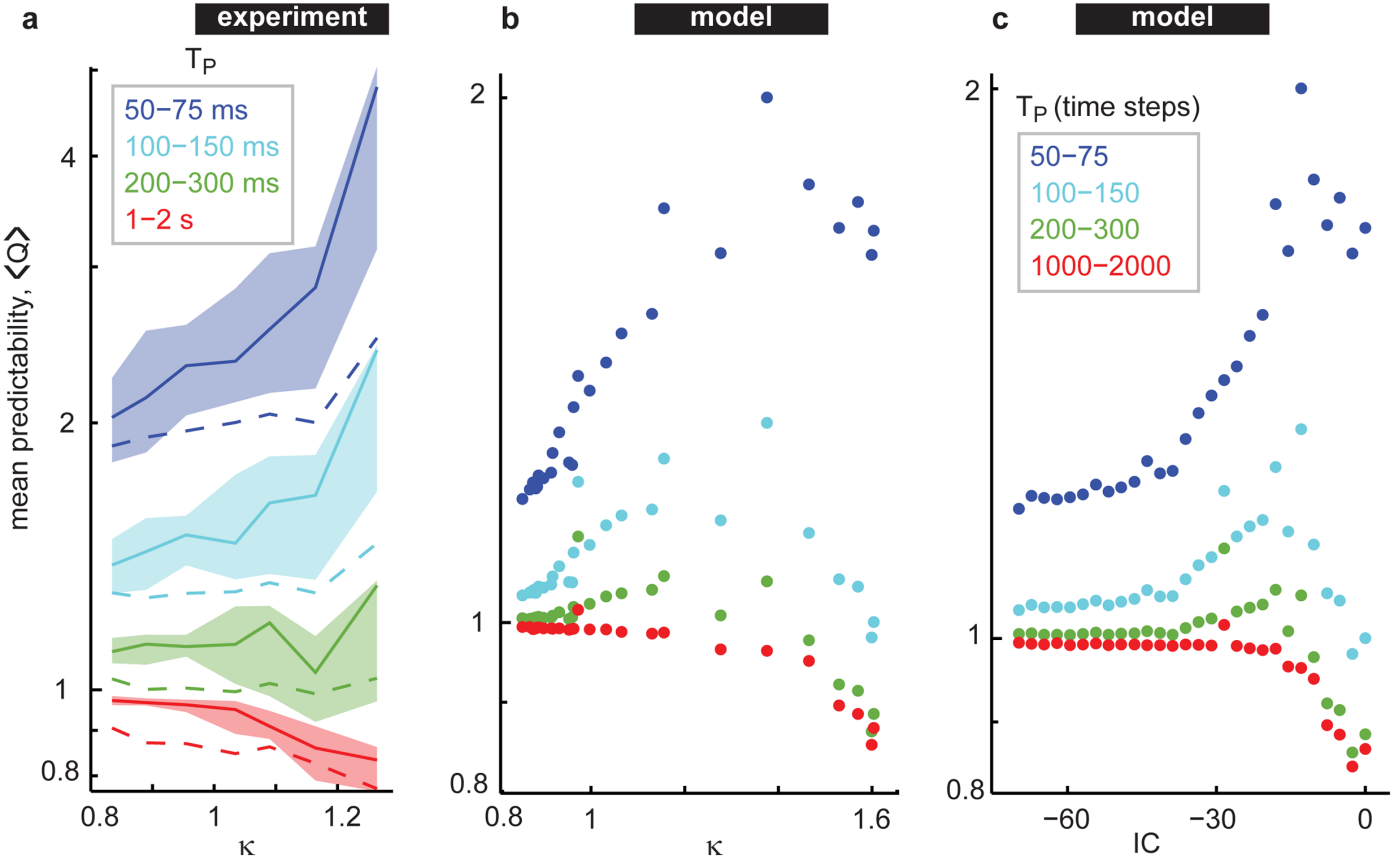
Time-dependent reversal of predictability vs. cortical state. **a)** For short-term predictions (T_P_ = 10–100 ms, purple), mean predictability rises as the cortical state is tuned from asynchronous to synchronous, i.e. for increasing κ. For longer-term predictions (T_P_ = 1–2 s, red), this trend reverses; low κ is more predictable than high κ. Each curve summarizes data from all experiments (n = 72). Solid lines indicate the median. Shaded region delineates quartiles. Dashed lines represent the mean control data obtained by phase shuffling the LFP before computing predictions. Vertical axes are logarithmic. **b)** We observed the same trend in the model for the experimentally observed range of states (0.8 > κ > 1.3). For more extremely synchronous states (κ > 1.3) the model revealed a decline in predictability for all prediction durations. **c)** The model data predictability values are shown versus inhibitory synapse strength *IC*. The <Q> values in this figure (all panels) represent an average of Q over all T_F_, all T_RES_, and a range of T_P_ for each dataset.

Finally, we determined how predictability depends on the cortical state, i.e. how Q depends on κ for broadband LFP (1–100 Hz) both in the experiment and the model (Fig 5). In our experiments, we found that for short time predictions (small T_P_), Q rises gradually as κ is increased from 0.7 to near 1 and then increases more sharply for κ>1. For longer time predictions (large T_P_), we were surprised to find precisely the opposite trend; Q falls gradually as κ is increased from 0.7 to near 1 and then decreases more rapidly for κ>1. However, all predictions for large T_p_ were rather poor. More quantitatively, we found that for 50 ms < T_P_ < 75 ms, Q and κ were correlated with Spearman’s ρ = 0.44 (p < 10^−4^).

To better understand the sources and significance of this relationship, we did a control analysis based on phase shuffling LFP before computing Q. Phase shuffling was performed independently for each electrode, thus destroying inter-electrode correlations, but preserving the basic character of fluctuation on each electrode. These control Q values (dashed lines in Fig 5a) were insignificantly correlated with κ (ρ = 0.16, p<0.2), for 50 ms < T_P_ < 75 ms. At longer timescales, 1 s < T_P_ < 2 s, real Q and κ were strongly anticorrelated (ρ = -0.59, p < 10^−7^), while control Q was uncorrelated (ρ = -0.22, p < 0.1). For intermediate timescales, 100 ms < T_P_ < 150 ms, real Q and κ were correlated (ρ = 0.30, p < 0.01), while control Q was uncorrelated with κ for this T_P_ range (ρ = 0.004, p < 0.9). For 200 ms < T_P_ < 300 ms, neither the real Q nor control Q was correlated with κ. The fact that phase shuffling the LFP before computing predictions destroyed the trends in Q vs κ for short and long time scales indicates that these trends cannot be explained by considering basic single-electrode properties of LFP fluctuations (amplitude, power spectrum, autocorrelation) across different cortical states. Indeed, phase shuffling preserves these properties at the single-electrode level. Moreover, since our phase shuffling control destroys inter-electrode correlations, inter-electrode interactions must play a role in determining state-dependence of predictability, rather than simpler prediction based on an electrode’s own history.

These data demonstrate that the highly synchronous state with κ>1 is relatively difficult to predict at long times, but quite predictable at short times. This variability in predictability across timescales is less dramatic for asynchronous cortical states with κ<1. Moreover, the mean predictability across timescales is highest in the synchronous state and lowest in the asynchronous state. A balance is found for intermediate states with κ~1; the mean predictability is not too low, and the variability in predictability is not too high. These experimental results were in good agreement with our model results, but the model allowed us to extend the range of κ values to higher values. For these extremely synchronous states, the model revealed a drop in predictability for the highest κ values (κ>1.3) even for short time predictions.

## Discussion

Here we measured multisite ongoing population activity in anesthetized rat somatosensory cortex. We showed that LFP can be predicted for short periods based on its own history using a simple autoregressive model, but that the efficacy of prediction depended sensitively on the cortical state and how far into the future the prediction was attempted. Based on distributions of population activation events, called neuronal avalanches, we parameterized a continuum of cortical states ranging from asynchronous, weakly correlated activity to large-scale synchronous activity. We found that near future (~10–100 ms) predictability is lower for the weakly correlated end of the continuum compared to synchronous states. This trend reverses for longer term predictions (~1 s); the synchronous state was less predictable than the asynchronous state. We observed a similar continuum of states and relationship between predictability and state in a network model of spiking neurons.

Previous studies have also addressed the predictability of ongoing cortical dynamics [14,21,22], usually with a goal of explaining trial-to-trial variability in response to sensory input. Our results suggest that such trial-to-trial variability may also depend on network state. We leave this interesting question open for future studies.

We parameterized the continuum of observed cortical states using κ, which measures how the avalanche size distribution deviates from a power law with exponent -1.5, as in previous studies [11,15,17,19,23]. Such power law distributed avalanches are predicted to occur at the critical point of a phase transition [24–26]. The hypothesis that cortical network dynamics can be tuned through a phase transition has a long history with origins in statistical physics research and, more recently, growing support from neuroscience experiments [19,27–31]. Near the ‘center’ of our observed continuum of cortical states, we found κ≈1, i.e. avalanche size distributions that were close to power law in form. This observation suggests that the continuum of cortical states we observed spans a critical phase transition, both in the experiment and the model. In this context, our observations suggest that, for both short and long time scales, criticality marks the tipping point between low to high predictability. However, which way the predictability tips, toward high or low values, depends on the time scale of prediction. Although many types of phase transitions can occur in different systems, all can be characterized as a transition between an ordered phase and a disordered phase. In the context of our findings, the disordered phase corresponds to the asynchronous, low kappa end of the cortical state continuum. The ordered phase corresponds to the synchronous, high kappa end of the continuum. From this point of view, it is perhaps not surprising that the ordered phase is more predictable than the disordered phase, as we see for the short term predictions. However, the drop in predictability in the ordered phase for longer time scales is more surprising.

One limitation of our work is that the continuum of cortical states was observed in anesthetized animals. Thus, it remains for future experiments to test whether a similar continuum of cortical states and corresponding predictability exists during wakefulness. This possibility is plausible, because previous studies have demonstrated behaviorally relevant changes in κ. For example, EEG recordings in humans suggest that sleep deprivation can elevate κ [32], while increased attention to a reaction time task can decrease κ [33]. Another study has shown that as a mouse awakens following pentobarbital anesthesia, κ decreases from values typically >1 to values closer to 1 as the arousal increases [19]. In this context, our results predict that the extremes of predictability, either high or low, are avoided in the awake state. We anticipate that future experiments will provide answers to these interesting questions.

## Materials and methods

### Electrophysiology

All procedures were carried out in accordance with the recommendations in the Guide for the Care and Use of Laboratory Animals of the National Institutes of Health and approved by University of Arkansas Institutional Animal Care and Use Committee (protocol #12025). We studied adult male rats (n = 12, 328±54 g; Rattus Norvegicus, Sprague-Dawley outbred, Harlan Laboratories, TX, USA). Anesthesia was induced with isoflurane inhalation and maintained with urethane (1.5 g/kg body weight (bw) dissolved in saline, intraperitoneal injection (ip)). Dexamethasone (2 mg/kg bw, ip) and atropine sulphate (0.4 mg/kg bw, ip) were administered before performing a 2 mm x 2 mm craniotomy over barrel cortex (1 to 3 mm posterior from bregma, 5 to 7 mm lateral from midline).

Extracellular voltage was recorded using 32-channel microelectrode arrays (8 shanks, 4 electrodes/shank, 200 μm inter-electrode distance, 400 μm inter-shank distance, A468-5mm-200–400-177-A32, NeuroNexus, MI, USA). Insertion depth was 650 μm, centered 2 mm posterior from bregma and 6 mm lateral from midline. Voltages were measured with respect to an Ag/AgCl ground pellet placed in the saline-soaked gel foams, which protect the exposed tissue surrounding the insertion site. Voltages were digitized with 30 kHz sample rate (Cereplex + Cerebus, Blackrock Microsystems, UT, USA). Recordings were filtered between 300 and 3000 Hz and thresholded at -3 SD to detect multi-unit activity (MUA).

### Pharmacology

Six 20 min recordings were conducted with each rat. First, three recordings were performed with no direct manipulation of inhibition (n = 36, indirect effects may be imposed by anesthetics [34] and atropine sulfate). Then, three recordings were performed with a drug topically applied via gel foam pieces soaked in saline mixed with drug. Three drug conditions were studied (one condition per rat): 1) 20 μM muscimol (6 rats, 18 recordings), 2) 20 μM bicuculline methiodide (3 rats, 9 experiments), 3) 40 μM bicuculline methiodide (3 rats, 9 experiments).

### Avalanche definition

We define an avalanche based on the spike count time series *c*(*t*) of MUA recorded on all electrodes, counting spikes in consecutive 15 ms time bins. An avalanche begins at t_i_ when c(t_i_) exceeds a threshold. The avalanche ends at t_f_ when c(t_f_) drops back below the threshold. The threshold is defined for each recording as the time average of c(t). The avalanche size *s* is defined as the total number of spikes occurring between t_i_ and t_f_, $s=\sum_{n=i}^{f}c\left(t_{n}\right)$. Our approach for defining avalanches contrasts with many previous experimental studies of neuronal avalanches, which were mostly based on local field potential. Here we sought to define avalanches based on spiking activity for two reasons. First, spike activity is easier to interpret than local field potential. Second, most theoretical and model studies of neuronal avalanches are based on spikes. A few other experimental studies have considered spike avalanches, but defined the start and stop of avalanches as time periods with no spiking [35,36]. In our view, this previous definition is inherently limited, because it does not scale well with increasing numbers of measured neurons and does not account for the fact that some neurons fire at extremely high rates. More specifically, if sufficiently many neurons are measured, periods with no spiking will become arbitrarily short. The definition of an avalanche should not be limited in this way. Moreover, it may be the case that the cortex operates in a regime with inherently self-sustained ceaseless dynamics, as studied recently in the context of avalanches [16]. In this case, it does not make sense to define avalanches based on quiet periods with no spiking, because such quiet periods do not exist. As tools for measuring large populations of neurons become more prevalent, it is likely that a well-sampled population of neurons in real cortex will also be devoid of meaningful silent periods.

### κ parameter

Deviation from the reference power-law (-1.5 exponent) was quantified with κ, which is a previously developed non-parametric measure with similarities to a Kolmogorov-Smirnov statistic [17,19,23]; κ equals 1 plus the sum of 10 differences between the observed avalanche size distribution (recast as a cumulative distribution) and a perfect power-law with exponent -1.5 (in cumulative form). The differences were computed at 10 sizes logarithmically spaced between the minimum and maximum observed avalanche sizes.

### Computational model

Our model consisted of 2952 spiking neurons– 80% excitatory, 20% inhibitory—placed on a 72 x 36 grid (the 2:1 aspect ratio matched the geometry of the experimental electrode arrays). Each neuron was modeled with two coupled differential equations which were derived from the Hodgkin—Huxley equations by Izhikevich [12],

$$\frac{{\text{dv}}_{\text{i}}}{\text{dt}}=0.04{\text{v}}_{\text{i}}^{2}+5{\text{v}}_{\text{i}}+140-{\text{u}}_{\text{i}}+{\text{I}}_{\text{i}}$$

$$\frac{{\text{du}}_{\text{i}}}{\text{dt}}=\text{a}({\text{bv}}_{\text{i}}-{\text{u}}_{\text{i}}),$$

where *v*_*i*_ represents the membrane potential of neuron *i* and *u*_*i*_ represents the ‘recovery’ variable of neuron *i*. The parameter *a* represents a time scale of recovery and *b* couples the membrane potential and recovery potential. For all excitatory neurons, *a* = 0.02 and *b* = 0.2. For inhibitory neurons *a* and *b* are drawn from uniform distributions on [0.02, 0.1] and [0.2, 0.25], respectively. When the membrane potential *v*_*i*_ exceeds 30, the neuron fires. Upon firing, *v*_*i*_ is reset to -65 and *u*_*i*_ is incremented to *u*_*i +*_ *d*, where *d* is 2 for all inhibitory neurons and drawn from a uniform distribution on [2, 8] for excitatory neurons. We use the numerical techniques developed by Izhikevich [12] to simulate these dynamics.

The input *I*_*i*_ to neuron *i* is comprised of random external input *η* and input due to presynaptic firing of the other neurons in the model,

$$I_{i}\left(t\right)=\eta_{i}\left(t\right)+\sum_{j\;=\;1}^{N}S_{ij}x_{j}(t-1),$$

Where *η* is drawn from a uniform distribution with mean 3 and unity standard deviation. Here *x*_*j*_(*t*) is a binary variable equal to one when neuron j fires and zero otherwise. The synapse from neuron j to neuron i is represented by the connection matrix element *S*_*ij*_. The *S* matrix was constructed in three steps. First, for excitatory presynaptic neurons, *S*_*ij*_ is drawn from a normal distribution with mean 8.4 and standard deviation 1.5. Second, for inhibitory presynaptic neurons, *S*_*ij*_ is drawn from a normal distribution with mean *IC* and standard deviation 1.5. To model the experimental pharmacological manipulation of inhibitory interactions we studied 30 different values of *IC* linearly spaced between -75 and 0. Finally, long-range connections were attenuated, by multiplying all *S*_*ij*_ by a distance dependent factor $e^{-d_{ij}^{2}/d_{o}^{2}}$, where *d*_0_ = 2.24 and *d*_*ij*_ is the distance between neuron i and neuron j. For each *IC*, the model was run for 500000 time steps (500 s, considering 1 time step to be 1 ms).

### Model LFP

To derive an LFP-like variable from our model dynamics we divided the 2D grid into 32 equal groups (each 9 x 9 in model grid space) meant to represent the 32 electrode channels in our experiments. To obtain the LFP for each group, we computed the average *v*_*i*_ across all neurons within the group, in line with experiments that show a close relationship between membrane potential fluctuations and LFP [37]. We clipped spikes before this averaging process. Finally, we filtered the model LFP just as we did with the experimental data.

### Auto regressive model fitting and prediction

We use a first order autoregressive model to generate the predictions reported here. The model specifies how each single LFP channel at time t, y_i_(t), is determined by all other LFP channels at previous times

$${\text{y}}_{\text{i}}\left(\text{t}\right)=\sum_{\text{j = 1}}^{n}{\text{A}}_{\text{ij}}{\text{y}}_{\text{j}}\left(\text{t}-1\right)+\epsilon (\text{t}),$$

where n is the number of LFP channels (32 in our experiments and model) and t is a discrete variable advancing 1 per sample. A is a 32 x 32 matrix which specifies how each channel influences each other channel and ∈ is a noise term. The matrix A and the noise ∈ are determined by fitting this model to a short period T_f_ of recorded data. For the fitting procedure, we used the algorithm developed by Neumaier and Schneider [38] and implemented with the ‘ARfit’ functions developed by Tapio Schneider for Matlab (Mathworks). After obtaining the best fit *A* based on the period starting at time t and ending at time *t+T*_*f*_, we then constructed a prediction over a time period with duration *T*_*p*_ starting at time *t+T*_*f*_*+1*. The prediction is constructed by iteratively applying the above equation starting with y(t + T_f_ + 1) as an initial condition. We define the prediction quality Q = X/dX, where X is the L1 norm of the actual measured data during the prediction time period

$$X=\sum_{\tau \;=\;t+T_{f}+1}^{t\;+\;T_{f}\;+\;T_{p}}\left|y_{real}\left(\tau \right)\right|$$

and dX is the L1 norm of the difference between the prediction and the read data

$$dX=\sum_{\tau \;=\;t+T_{f}+1}^{t\;+\;T_{f}\;+\;T_{p}}|y_{real}\left(\tau \right)-y_{predicted}\left(\tau \right)|.$$

Thus, Q less than or near 1 indicates a poor prediction while Q>>1 indicates a good prediction.

## Supporting information

S1 File We include the ARRIVE checklist for enhancing reproducibility of research. (DOCX)

S2 File We include a related manuscript [17], which was based on the same experimental data analyzed here. (PDF)
