# Supplementary material for: State-dependent intrinsic predictability of cortical network dynamics
Source: PLoS One. 2017 May 4;12(5):e0173658. doi: 10.1371/journal.pone.0173658 (PMC5417414; doi:10.1371/journal.pone.0173658)

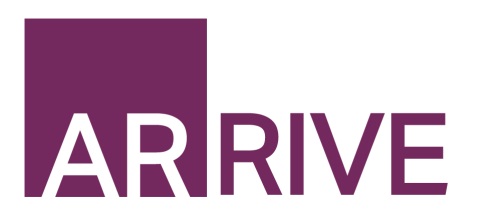


The ARRIVE Guidelines Checklist

Animal Research: Reporting In Vivo Experiments

Carol Kilkenny^1^, William J Browne^2^, Innes C Cuthill^3^, Michael Emerson^4^ and Douglas G Altman^5^

*^1^The National Centre for the Replacement, Refinement and Reduction of Animals in Research, London, UK, ^2^School of Veterinary Science, University of Bristol, Bristol, UK, ^3^School of Biological Sciences, University of Bristol, Bristol, UK, ^4^National Heart and Lung Institute, Imperial College London, UK, ^5^Centre for Statistics in Medicine, University of Oxford, Oxford, UK.*

|  | | ITEM | RECOMMENDATION | Section/ Paragraph |
| --- | --- | --- | --- | --- |
| 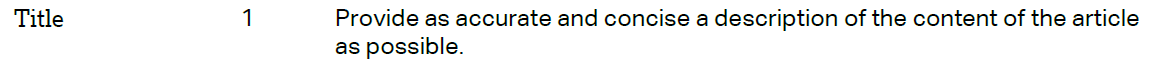 | | | Title |  |
| 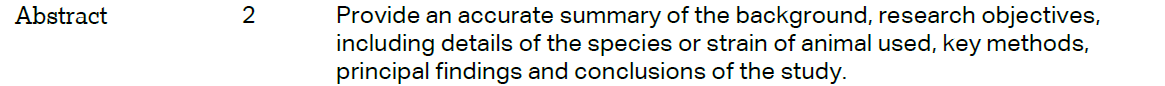 | | | Abstract |  |
| INTRODUCTION | | |  |  |
| 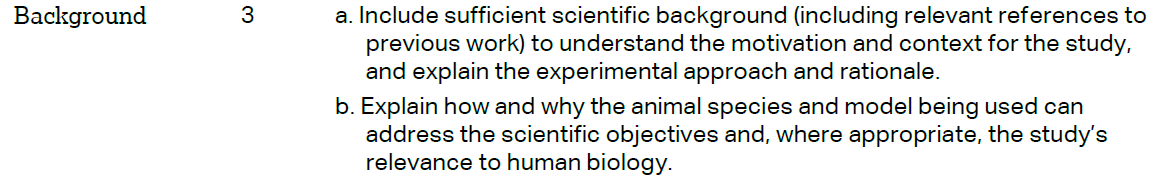 | | | Introduction / Paragraph 1 |  |
| 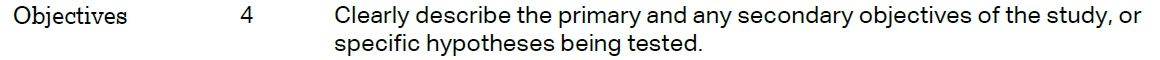 | | | Introduction/Paragraph 1 |  |
| METHODS | | |  |  |
| 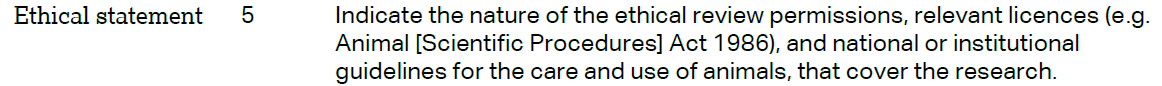 | | | Materials and Methods / Paragraph 1 |  |
| 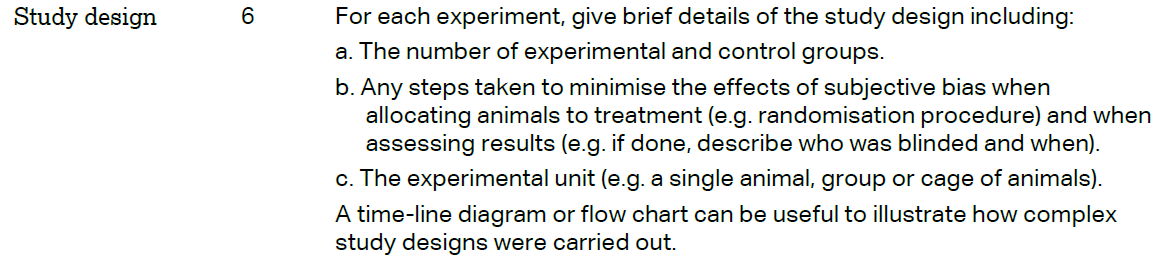 | | | Materials and Methods / Paragraph 1 & 2 |  |
| 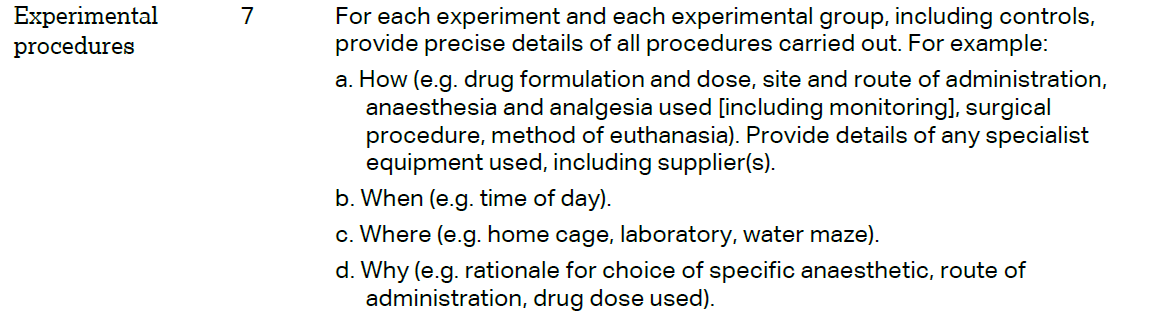 | | | Materials and Methods / Paragraph 1 & 2 |  |
| 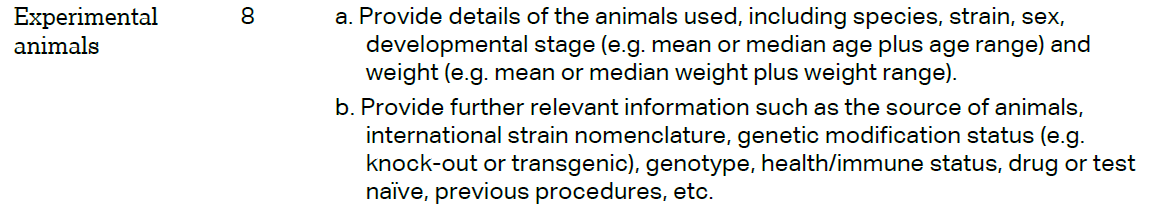 | | | Materials and Methods / Paragraph 1 & 2 |  |

The ARRIVE guidelines. Originally published in *PLoS Biology*, June 2010^1^

| 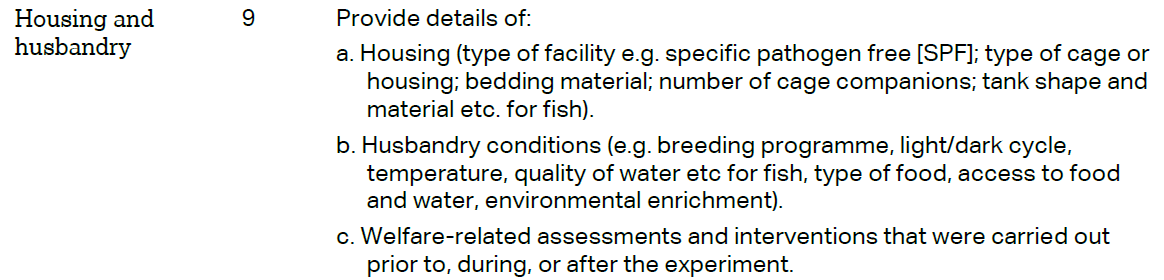 | Materials and Methods / Paragraph 1 & 2 | |
| --- | --- | --- |
| 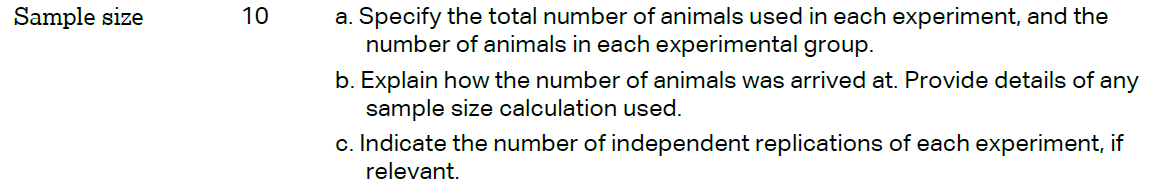 | Materials and Methods / Paragraph 1 & 2 | |
| 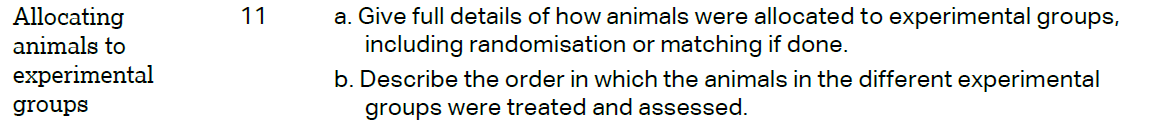 | Materials and Methods / Paragraph 1 & 2 | |
| 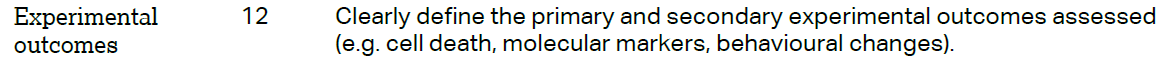 | Materials and Methods / Paragraph 3 & 4 | |
| 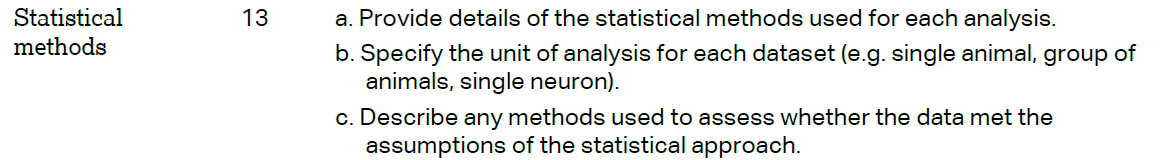 | Materials and Methods / Paragraph 3 & 4 | |
| RESULTS |  | |
| 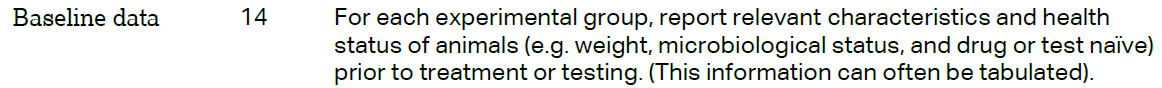 | Materials and Methods / Paragraph 1 & 2 | |
| 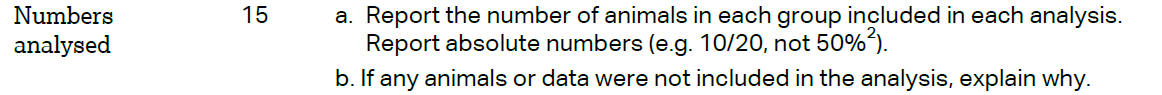 | Materials and Methods / Paragraph 1 & 2 | |
| 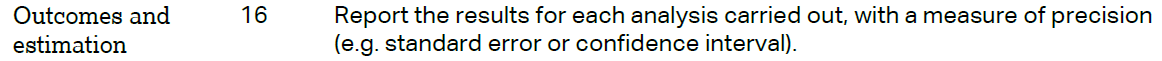 | Results / Paragraphs  1-8 | |
| 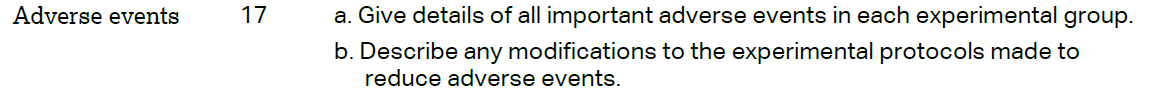 | n/a | |
| DISCUSSION |  | |
| 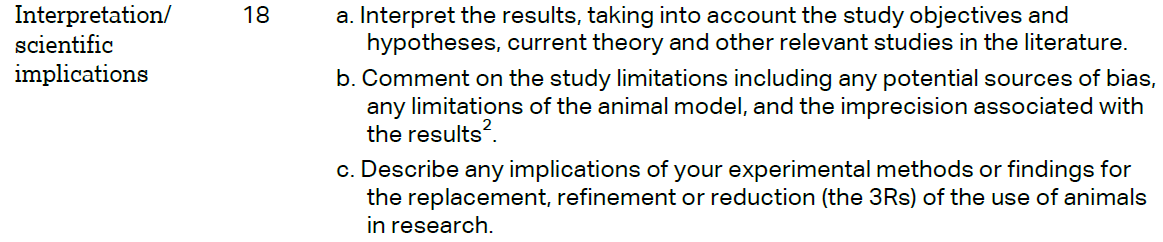 | Discussion / Paragraph 1 - 4 | |
| 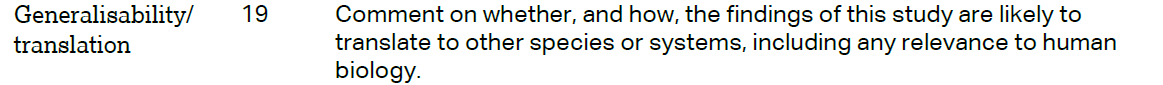 | Discussion / Paragraph 1 - 4 | |
| 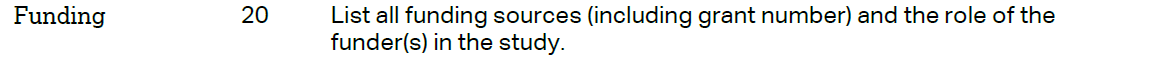 | | Funding Statement |


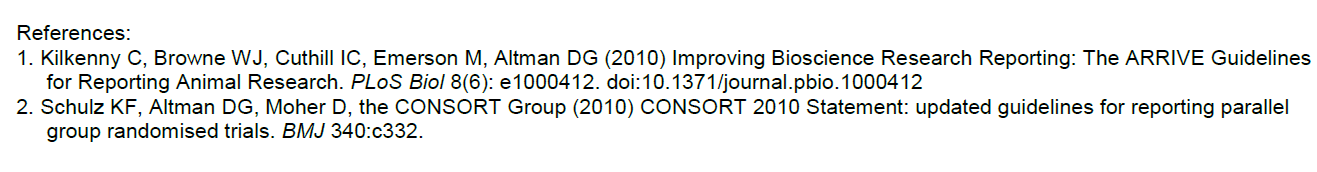

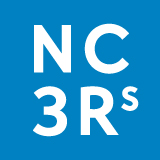

Supplement: S1 File — (DOCX) [file pone.0173658.s001.docx]
