# Supplementary material for: State-dependent intrinsic predictability of cortical network dynamics
Source: PLoS One. 2017 May 4;12(5):e0173658. doi: 10.1371/journal.pone.0173658 (PMC5417414; doi:10.1371/journal.pone.0173658)
Supplement: S2 File — (PDF) [file pone.0173658.s002.pdf]

PLOS COMPUTATIONAL BIOLOGY

RESEARCH ARTICLE

Maximizing Sensory Dynamic Range by Tuning the Cortical State to Criticality

Shree Hari Gautam, Thanh T. Hoang, Kylie McClanahan, Stephen K. Grady, Woodrow L. Shew*

Department of Physics, University of Arkansas, Fayetteville, Arkansas, United States of America

* shew@uark.edu

CrossMark
click for updates

OPEN ACCESS

Citation: Gautam SH, Hoang TT, McClanahan K, Grady SK, Shew WL (2015) Maximizing Sensory Dynamic Range by Tuning the Cortical State to Criticality. PLoS Comput Biol 11(12): e1004576. doi:10.1371/journal.pcbi.1004576

Editor: Claus C. Hilgetag, Hamburg University, GERMANY

Received: July 27, 2015

Accepted: September 20, 2015

Published: December 1, 2015

Copyright: © 2015 Gautam et al. This is an open access article distributed under the terms of the Creative Commons Attribution License, which permits unrestricted use, distribution, and reproduction in any medium, provided the original author and source are credited.

Data Availability Statement: Data are available for download from http://www.woodrowshew.com/home/whisker-project-data.

Funding: WLS and SHG were funded in part by the Arkansas Biosciences Institute (Grants 0055 and 0070; http://arbiosciences.org/). The funders had no role in study design, data collection and analysis, decision to publish, or preparation of the manuscript.

Competing Interests: The authors have declared that no competing interests exist.

Abstract

Modulation of interactions among neurons can manifest as dramatic changes in the state of population dynamics in cerebral cortex. How such transitions in cortical state impact the information processing performed by cortical circuits is not clear. Here we performed experiments and computational modeling to determine how somatosensory dynamic range depends on cortical state. We used microelectrode arrays to record ongoing and whisker stimulus-evoked population spiking activity in somatosensory cortex of urethane anesthetized rats. We observed a continuum of different cortical states; at one extreme population activity exhibited small scale variability and was weakly correlated, the other extreme had large scale fluctuations and strong correlations. In experiments, shifts along the continuum often occurred naturally, without direct manipulation. In addition, in both the experiment and the model we directly tuned the cortical state by manipulating inhibitory synaptic interactions. Our principal finding was that somatosensory dynamic range was maximized in a specific cortical state, called criticality, near the tipping point midway between the ends of the continuum. The optimal cortical state was uniquely characterized by scale-free ongoing population dynamics and moderate correlations, in line with theoretical predictions about criticality. However, to reproduce our experimental findings, we found that existing theory required modifications which account for activity-dependent depression. In conclusion, our experiments indicate that *in vivo* sensory dynamic range is maximized near criticality and our model revealed an unanticipated role for activity-dependent depression in this basic principle of cortical function.

Author Summary

When many simple parts interact, the collective behavior of the whole can be astonishingly complex. A particularly striking example is our capacity for sensory perception, which results from the collective interactions of billions of relatively simple neurons. Another example is found in physical systems which undergo a phase transition–for example, liquid water turning to solid ice. When collective interactions among the water molecules are changed, the system transitions from a disordered state (liquid) to an ordered state (crystalline solid). At the tipping point of a critical phase transition, i.e. at criticality, physical

systems exhibit very complex behavior. In this study, we show that phase transitions may occur in the cerebral cortex changing the neural activity from a disordered to an ordered state. Moreover, this neural phase transition may be intimately linked with sensory perception. We experimentally manipulate the interactions among neurons and show that sensory dynamic range is maximized when the cerebral cortex of a rat is closest to criticality.

Introduction

Cortical neuronal network dynamics shift among myriad states to cope with the changing needs of the organism [1–3]. Strikingly different cortical states are observed during different behaviors such as sleep [4], wakeful resting [5], active movement [6], or vigilant attention [7]. Externally-imposed manipulations of interactions among cortical neurons, like neuromodulators [7–9], anesthetics [10–12], and other drugs [13,14], also alter the cortical state. Which cortical states are optimal for gathering information about the world through sensory input? Answers to this question are only beginning to be understood. For example, previous studies have shown that changes in cortical state impact sensory adaptation [5], variability of cortical response [9,12,15,16], and the ability to track fast stimulus changes [12,17]. Here we focused on the ability of cortical neuronal networks to distinguish a wide range of stimulus intensities, i.e. sensory dynamic range. We sought to delineate how sensory dynamic range depends on cortical state. To meet this goal, we took advantage of changes in cortical state that occur naturally [15,18] without experimental control and we also imposed changes in cortical state by tuning cortical inhibitory interactions [19].

Our approach was motivated, in part, by theory [20–22] and in vitro experiments [19] which point to a potential general principle governing cortical dynamic range. They proposed that dynamic range is maximized by tuning the cortex to operate at criticality. Criticality is a boundary regime separating two distinct regimes of cortical state [23,24]. On one side of the critical boundary, the ‘subcritical’ cortical state is characterized by asynchronous population activity, low firing rates, and low sensitivity to stimuli. On the other side, the ‘supercritical’ cortical state is marked by large-scale, coordinated population activity and tends to be hyperexcitable in response to stimulation. Cortical dynamic range is thought to be low in the subcritical state due to insensitivity to weak stimuli. In contrast, existing theory suggests that dynamic range is low in the supercritical regime because the system tends to saturate with most neurons in the network firing at high rates, even without external input. Criticality is thought to be optimally balanced between these extremes, able to detect weak stimuli without saturating. However, this potentially fundamental relationship between criticality and sensory dynamic range has not been tested in an intact sensory system. Indeed, the theory may be irrelevant because in vivo cortical networks never reach the saturated firing regime that has been theoretically shown to be responsible for low dynamic range in the supercritical state. Synaptic depression and other mechanisms serve to prevent such saturated firing. Thus, it remains unclear if in vivo sensory dynamic range will indeed be highest when the cortex operates near criticality.

Here we directly measured the in vivo relationship between cortical state and somatosensory dynamic range in the rat whisker system. We found that dynamic range is highest in cortical states that exhibit signs of criticality. However, our experimental observations were not well-explained by existing theories, particularly in the experimentally observed supercritical regime. To account for our experimental results we used a model with strong activity-dependent depressive effects, thus avoiding the saturated response in the supercritical regime. Thus, we

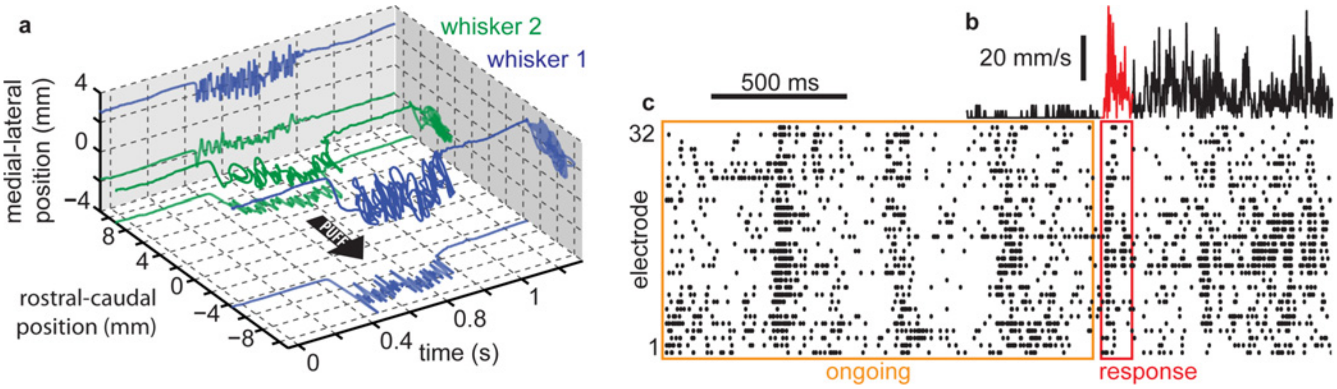

Fig 1. Ongoing population spiking activity and response to whisker stimulation. (a) Simultaneously recorded two-dimensional motion of two whiskers during an air puff stimulus. **(b)** Speed of dominant whisker during recording shown in (c). **(c)** Multi-unit activity recorded from 32 electrodes in barrel cortex of an anesthetized rat. Ongoing activity (orange box), recorded while whiskers are stationary, was used to assess the cortical state. Population spike count was compared to average whisker speed during the first 100 ms after stimulus onset (red) to assess sensory dynamic range.

doi:10.1371/journal.pcbi.1004576.g001

conclude that, for reasons not anticipated by previous theory, in vivo sensory dynamic range is maximized near criticality.

Results

We investigated relationships among cortical state, inhibition, and sensory dynamic range in the whisker system of urethane anesthetized adult male rats (n = 13 rats, 94 recordings). We recorded multi-unit activity (MUA) in barrel cortex using 32-channel microelectrode arrays (Fig 1). Each rat was studied first in the normal anesthetized state, second, with pharmacologically altered synaptic inhibition, and, finally, under a wash condition. Changes in cortical state occurred for two reasons. First, we manipulated the cortical state by pharmacological modulation of synaptic inhibition, locally in the recorded region of cortex, by topical application of muscimol (GABA agonist) or bicuculline methiodide (GABA antagonist). Second, we found that changes in cortical state occurred naturally, without altered inhibition. Rather than 'averaging out' this natural variability of cortical state, we took advantage of it; we systematically parameterized the range of cortical states that we observed. This approach acknowledges that the cortical state is not static even under 'normal' conditions and state changes can result in significant experiment-to-experiment and animal-to-animal variability. During times with no whisker stimulus, we quantitatively assessed the cortical state using multiple features of ongoing MUA activity, including correlations, spatiotemporal variability, and the prevalence of different spatiotemporal scales. We analyzed the response to stimulation to quantify sensory dynamic range.

Parameterizing the cortical state based on spatiotemporal scales of population activity

First, we parameterized the cortical state based on the prevalence of different spatiotemporal scales of population spiking activity. Our approach accounts for the relative importance of diverse scales, avoiding bias for any particular scale. Motivated by previous studies of spatiotemporal cascades of population activity called 'neuronal avalanches' [25–27], we began by making a population MUA spike count time series including spikes recorded on all electrodes. Then, 'avalanches' were defined as periods of time when the MUA spike count exceeded a threshold (Fig 2A). We note that our results were robust to variation (up or down by a factor of

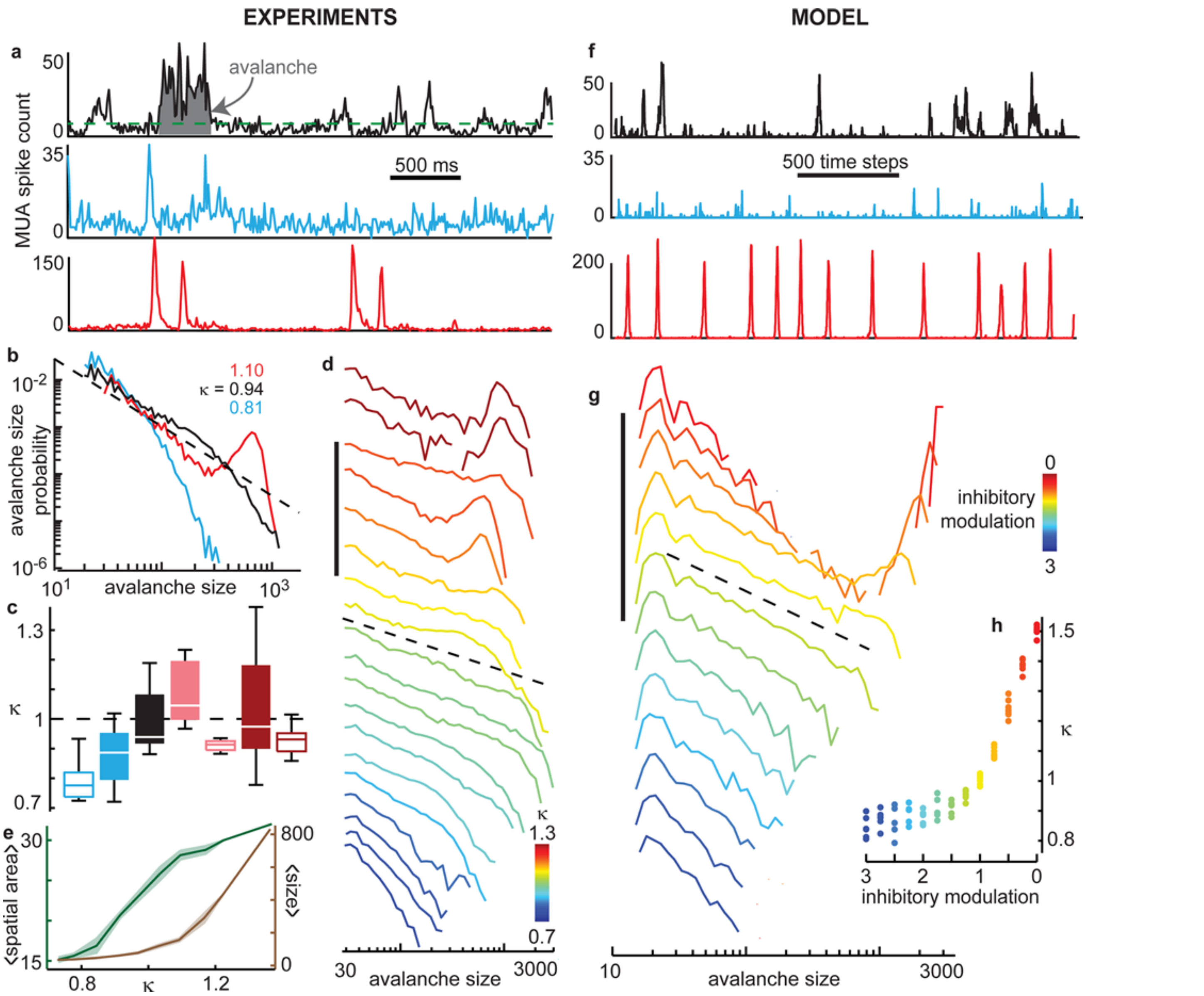

Fig 2. The cortical state is a tunable continuum. (a) Example MUA spike count time series during ongoing activity for unaltered inhibition (black), enhanced inhibition (blue), reduced inhibition (red). Green dashed line indicates threshold for defining avalanches. Shaded gray area indicates the size (total spike count) of one example avalanche. **(b)** Avalanche size distributions reveal that enhancing inhibition leads to a cortical state with predominantly small-scale activity (blue). Reducing inhibition leads to prominent large-scale activity (red). Unaltered inhibition results in diverse spatiotemporal scales (black), often distributed similar to a power law with -1.5 exponent (dashed). The parameter κ measures deviation from the power law. **(c)** Increasing inhibition tends to decrease κ and decreasing inhibition tends to increase κ, but κ varied widely across experiments with fixed drug condition. Color indicates drug condition: 20 μM muscimol (blue), no drug (black), 40 μM bicuculline (pink), 20 μM bicuculline (red). Filled boxes indicate drug condition. Open boxes indicate post-drug wash condition. Box delineates quartiles, line marks median, and whiskers span range of data. **(d)** Example distributions illustrating the continuum of cortical states, parameterized by κ (color). Vertical axis is logarithmic as in (b) with the scale bar spanning 5 orders of magnitude. Curves are shifted vertically for clarity. The dashed line is a reference power law with exponent -1.5; it is placed near the distributions with κ nearest 1. Some of these examples are from experiments with drugs applied, some from experiments with no drug. **(e)** The average spatial area (number of electrodes) of avalanches undergoes a sigmoidal rise as κ increases (green). The average size of avalanches increases sharply as κ increases beyond κ = 1 (brown). The rise in size versus κ lags the rise in spatial area, because size also depends on the avalanche duration. To illustrate these trends, we present median (line) and quartiles (shaded areas) across all experiments with similar κ. **(f)** Time series from the model at criticality (black, inhibitory modulation at 1), in the subcritical regime (blue, inhibitory modulation at 3) and in the supercritical regime (red, inhibitory modulation at 0). **(g)** Example distributions illustrating continuum of different model states, parameterized by inhibitory modulation (color). Vertical axis is logarithmic as in (b) with the scale bar spanning 5 decades. Note that power law avalanche size distributions only occur when the model operates at criticality (yellow, inhibitory modulation at 1). The dashed line is a reference power law with exponent -1.5; it is placed near the distributions with κ nearest 1. **(h)** We measured κ based on model distributions. Inhibitory modulation (color) was inversely and monotonically related to κ.

doi:10.1371/journal.pcbi.1004576.g002

2) in the choice of threshold and time bin duration (S1 and S2 Figs). The 'size' of each avalanche was defined as the total number of spikes occurring during the avalanche. To determine the prevalence of different spatiotemporal scales, for each recording, we examined the distribution of avalanche sizes (Fig 2B and 2D).

Examining avalanche size distributions over all of our experiments, we found that a continuum of different network states occurred (Fig 2D). At one end of the continuum, distributions were bimodal, indicating that large-scale avalanches played a prominent role in the cortex dynamics. This situation often occurred for pharmacologically reduced inhibition (Fig 2B and 2C). At the opposite end of the continuum, we observed cortical states in which small scales were dominant, often occurring when inhibition was enhanced (Fig 2B and 2C). The cortical state varied continuously between these extremes (Fig 2C). Near the middle of the continuum, we observed highly diverse avalanches with heavy-tailed distributions [23,28] of avalanche size, close to a power-law distribution with exponent -1.5 (Fig 2D). To quantitatively index the observed continuum of cortical states, we employed the parameter κ, which measured the deviation between the observed avalanche size distribution and a power law with exponent -1.5, as in previous work [11,19,27]. In brief, large κ entailed a cortical state with strongly coordinated population activity, commonly sweeping across the entire recording area (Fig 2E). For small κ, population activity was weakly coordinated, typically confined to small spatial extents (Fig 2E). Separating these extremes, the cortical state with $\kappa = 1$ exhibited more diverse population activity with power law distributed spatiotemporal scales. Power law avalanche size distributions have additional significance because they are predicted to occur in a specific cortical state called 'criticality', as discussed in the introduction section. The particular power law exponent -1.5 is associated with a particular type (universality class) of criticality, namely, directed-percolation [29] and has also been studied in other excitable networks [30].

Cortical state transition from weak to strong correlations

The degree of correlations among cortical neurons plays an important role in population coding [31] and cortical state [1]. Our second approach for assessing the cortical state was based on pairwise correlations of MUA recorded at different electrodes. For this, we created MUA spike count time series for each electrode, excluding the times when whiskers were stimulated. Then, we computed the Pearson correlation coefficient between all pairs of electrodes. We found that correlations were closely related to our state index κ. The distribution of pairwise correlation coefficients was broadest for states near $\kappa = 1$ (Fig 3A and 3B), which reflects the diversity of avalanche sizes that occur in such states. States with either small or large κ had relatively narrow distributions of correlations with decreased or increased average correlations, respectively (Fig 3A and 3B). The average pairwise correlation of the population increased sigmoidally as κ is increased (Fig 3C). This demonstrates that the state with $\kappa = 1$ lies at the boundary separating two distinct dynamical regimes–one with low correlations, the other with high correlations.

Maximized dynamic range in a specific cortical state

Our ultimate goal was to determine how cortical somatosensory dynamic range depends on cortical state. For this, dynamic range was calculated based on average cortical neural response to a range of whisker stimulus intensities (Fig 4). We defined neural response to be the MUA spike rate during the 100 ms following stimulus onset (Figs 1 and 4A). We defined the stimulus to be the average whisker speed during the 100 ms following stimulus onset (Figs 1 and 4B). Repeated identical puff pressures generally resulted in different whisker motion. Therefore, we parameterized the stimulus based on measurements of the actual whisker speeds for each puff. Whisker speeds ranged from 0 to about 30 mm/s. For cortical states with small κ, the response curves

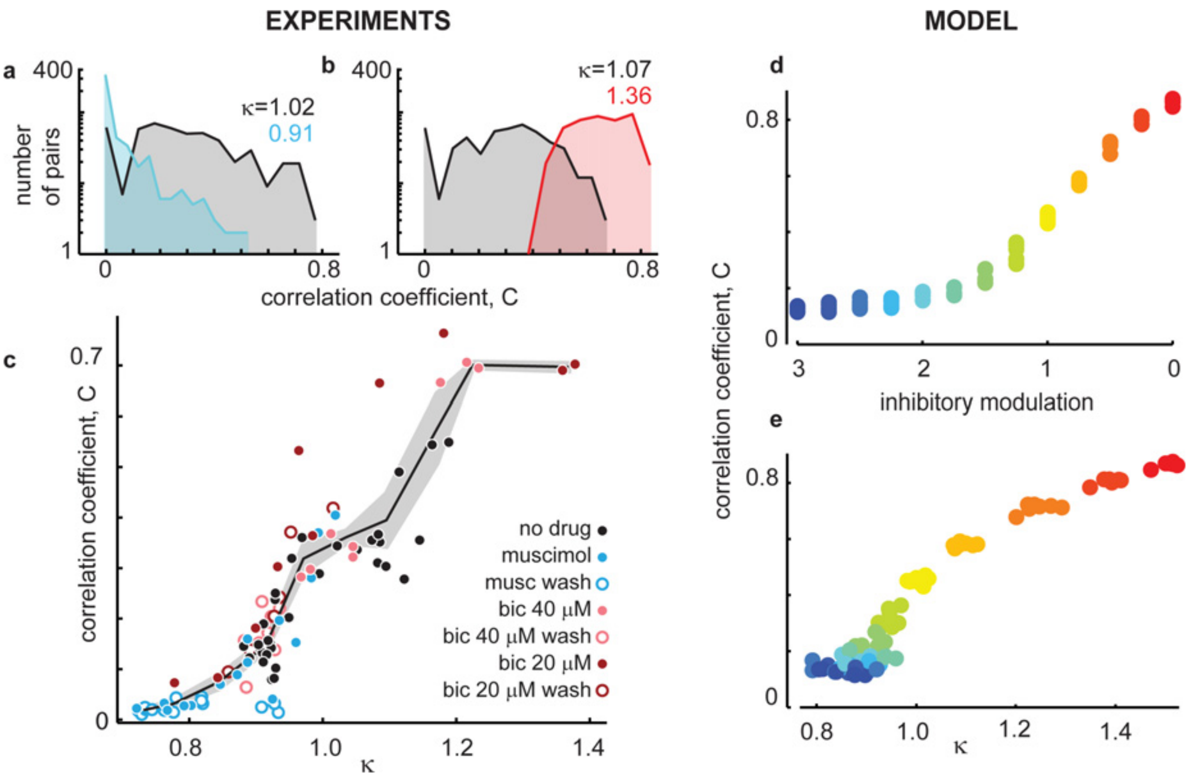

Fig 3. Continuum spans transition from weakly to strongly correlated cortical state. (a,b) Zero-lag Pearson correlation coefficients were computed for all electrode pairs (n = 496) during ongoing activity. Shown are example distributions of all pairwise correlations for unaltered inhibition (black), increased inhibition (blue), and decreased inhibition (red). Broader distributions for unaltered inhibition indicate that correlations are more diverse than for either increased or decreased inhibition. Data in (a) are from one rat; data in (b) is from another rat. **(c)** Mean pairwise correlations undergo a sigmoidal increase as κ increases, with κ = 1 dividing weakly correlated cortical states from strongly correlated states. To illustrate this trend, we computed the mean pairwise correlation for each experiment and present the mean (line) ± standard error (shaded areas) across all experiments with similar κ. **(d)** The model undergoes a similar transition from weak to strong population correlations as inhibition is tuned from strong to weak (blue to red, color map the same as Fig 2H). **(e)** Model results match experiments more closely when model state is parameterized by κ.

doi:10.1371/journal.pcbi.1004576.g003

tended to rise gradually with increasing whisker speed (Fig 4C). For states with large κ, the response curve tended to rise sharply and saturate for a relatively small whisker speed (Fig 4C). Dynamic range was defined based on the range of whisker speeds over which the response increased from 10% to 90% of the response range (Fig 4D, inset). The main result of our work is that dynamic range was low in experiments with either low κ or high κ; dynamic range was maximized for cortical states with κ≈1 (Fig 4D). We remind the reader that κ is based solely on ongoing activity; periods of stimulus-evoked activity are excluded when computing κ. Comparing dynamic range (Fig 4D) to correlations (Fig 3C), our results establish that cortical dynamic range is maximized for cortical states with an intermediate degree of correlations.

In the model, we obtained stimulus-response curves and dynamic range trends similar to those we observed in our experiments (Fig 4E). To obtain this match, we had to limit the range of stimulus intensities to be below 10^{-3} stimulus driven spikes per model time step. As discussed further below, further increases in stimulus intensity resulted in a further rise in the response curve and disagreement between the model and experiment.

Discussion

Our findings offer a specific answer to a long standing debate concerning what degree of correlations among neurons is optimal for sensory information processing [31–33]. If correlations

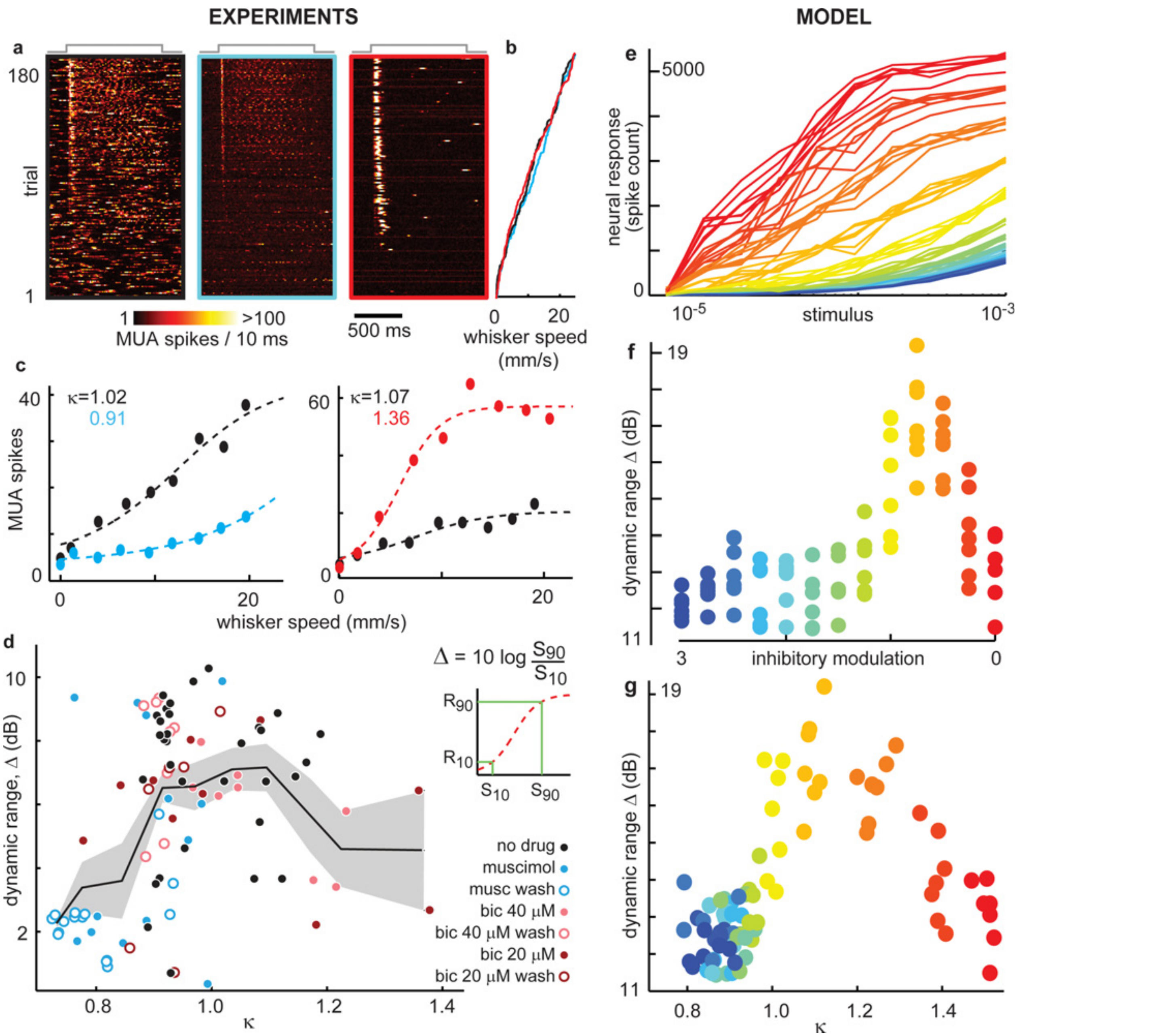

Fig 4. Peak dynamic range at intermediate cortical state near κ = 1. (a) MUA spike response for 180 trials of whisker stimulation ranked by stimulus strength (whisker speed) for unaltered (left), enhanced (middle), reduced inhibition (right). Color indicates total population MUA spike count. Gray line (top) indicates stimulus onset and duration. **(b)** Average speed of dominant whisker during 100 ms following stimulus onset for the 180 trials shown in (a). Color indicates inhibitory condition: none (black), enhanced (blue), or reduced (red). **(c)** Example stimulus-response curves. Typically, enhanced inhibition (blue) resulted in decreased sensitivity and gain compared to unaltered inhibition (black). Reduced inhibition (red) often increased gain so much that the response curve reached a saturated level for moderate stimuli. Each point represents the average MUA spike response conditioned on whisker speed during the first 100 ms following stimulus onset. Dashed line is a best-fit sigmoid curve. **(d)** Dynamic range Δ was highest for an intermediate cortical state close to κ = 1. (inset) Dynamic range was defined based on the best fit sigmoid. **(e)** Stimulus response curves from our model. Notice that the shapes of response curves change with inhibitory modulation (color code defined in panel f) as seen in experiments (panel c). **(f)** The highest dynamic range was found near criticality (with unmodulated inhibition). **(g)** Parameterizing the model state with κ reveals the model dynamic range depends on network state as seen experimentally (d).

doi:10.1371/journal.pcbi.1004576.g004

are too strong—many neurons firing synchronously—then coding is redundant and metabolically inefficient. At the other extreme, sufficiently weak correlations may compromise the robustness of information transfer among cortical circuits. Thus, functionally effective correlations must lie between these extremes, but pinpointing the optimal level of correlations has been an elusive goal. In the context of sensory dynamic range, our results demonstrate that the specific intermediate level of correlations that coincides with power law distributed avalanches ($\kappa = 1$) is optimal.

Our study was motivated by predictions of maximized dynamic range at criticality based on pioneering analytical and computational studies [20–22,34]. At first glance, these previous predictions appear to agree with our main findings here. However, taking a closer look, we found that these previous models and theories did not explain our experimental observations. The discrepancies were in the putative supercritical regime ($\kappa > 1$), experimentally observed when inhibition was suppressed. In this case, we observed large bursts of synchronous spiking activity occurring at irregular intervals, emerging from a mostly inactive baseline activity (red, Fig 2A). In contrast, in the supercritical regime of most previously studied models, ongoing activity manifests as persistent activity with nearly all neurons active at all times, with no quiet periods and no synchronous bursts (Fig 5A). We found that a simple way to modify previous models to produce more realistic, bursty dynamics was to introduce activity dependent depression–spiking probability was reduced in proportion to how may spikes occurred in the recent past (see Materials and Methods). This naturally resulted in large bursts of population activity separated by times of relative silence (Fig 2E and Fig 5A), as seen experimentally when inhibition was reduced. Without such depressive adaptation, our model produced sustained, saturated activity in the supercritical regime and dynamic range was maximized near criticality like in previous models (Fig 5A and 5B). Including depressive adaptation dramatically altered the shape of the stimulus response curve compared to that of a model without activity dependent depression (Fig 5B and 5C). In fact, if the full range of stimuli studied in previous models was considered, including those large enough to activate a large fraction of the network, then dynamic range was no longer maximized at criticality, as shown in Fig 5C. However, such large stimuli are not relevant in real sensory systems; even the most intense whisker stimulation does not result in a neural response that approaches the system size, i.e. all neurons firing. Thus, the most plausible comparison to our experiments was to exclude the large-stimulus section of the response curve. This limitation leads to response curves (Fig 4E) which match well with our experimental observations (Fig 4C) and, most importantly, recovers the result that dynamic range is maximized near criticality. In conclusion, our results indicate that a different mechanism than previously predicted is responsible for the experimental observation of peak somatosensory dynamic near criticality *in vivo*.

A natural question arises due to the fact that activity dependent depression dramatically changes the nature of network dynamics in the supercritical regime. Does activity-dependent depression change the nature of the phase transition; do we expect a continuous phase transition or some other type of phase transition? We leave this question to be answered by future theoretical work, but we speculate, based on the following reasoning, that the phase transition remains continuous. The activity-dependent depression has no effect on the model dynamics if spike rates are sufficiently low (< 1 spike per 80 time steps). Indeed, in the subcritical regimes where spike rates are relatively low, the stimulus response curves in Fig 5C (with depression) are not significantly different than those in Fig 5B (without depression). Since the tipping point of the phase transition is close to this low activity regime, it is likely that a continuous phase transition remains continuous when the model includes depression. This speculation is partially supported by the fact that our model produces power-law distributed avalanches (Fig 2G)

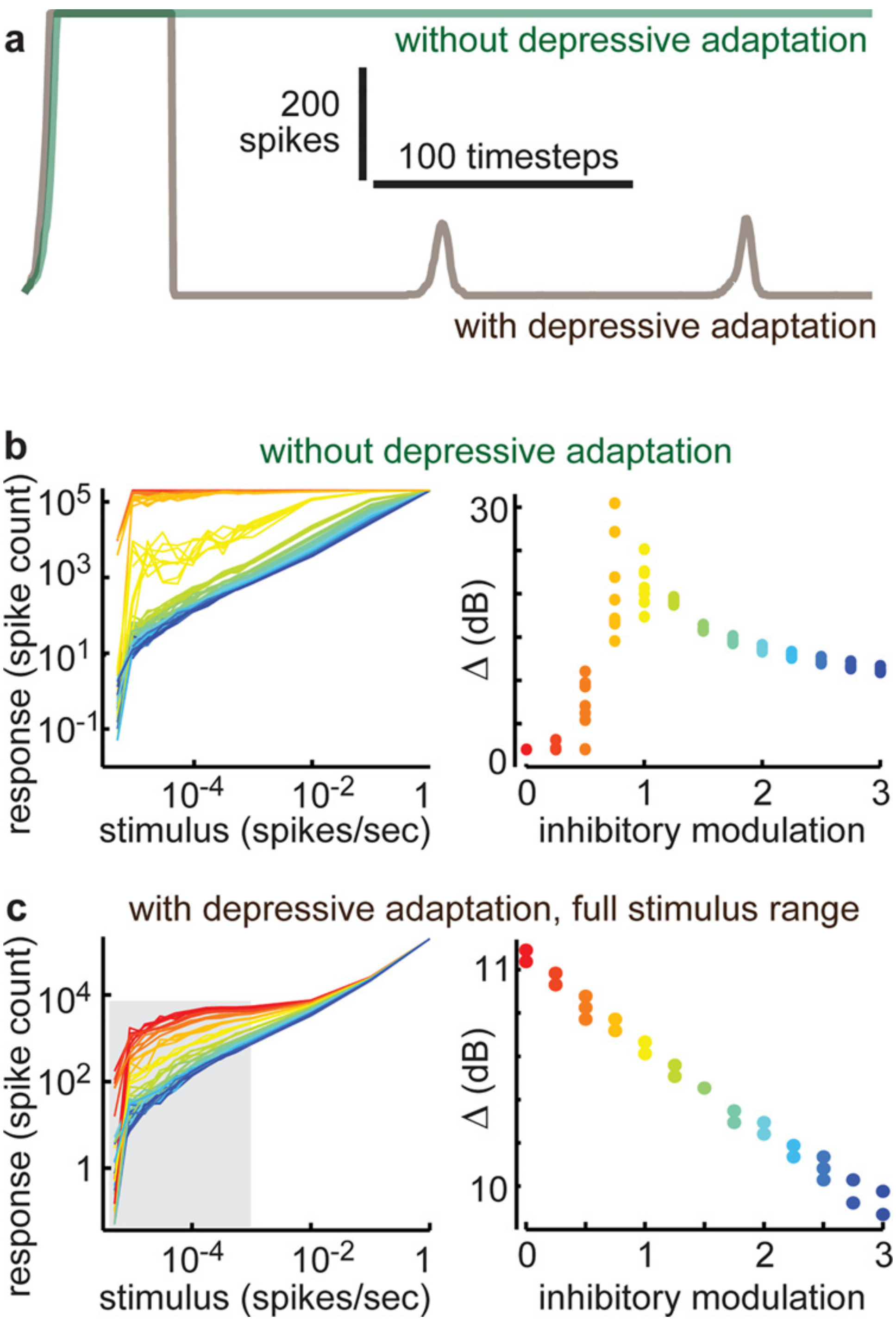

Fig 5. Explaining experiments requires model with activity dependent depression. Comparison of our model results with and without activity dependent depression. **a)** Without depression, the supercritical regime (inhibitory modulation at 0) results in sustained, saturated network activity (green). With depression, the system is quiet except during large bursts of activity, but never saturates (brown), similar to our experiments with suppressed inhibition (Fig 2A, red). **b)** As in previous studies without depression, dynamic range is maximized near criticality (inhibitory modulation near 1). Color indicates the inhibitory modulation factor as indicated by horizontal axis of right panel. **c)** With depression, the response curves change shape, decreasing for small stimuli (left). This change in shape mostly occurs in the supercritical regime (inhibitory modulation <1), leaving the critical and subcritical cases largely unchanged. This results in highest dynamic range for the supercritical state (right). However, if large stimuli are excluded (keeping the gray shaded

region), which is more realistic compared to our experiments, then dynamic range is again maximized at criticality, as shown in Fig 4E–4G. Color indicates the inhibitory modulation factor as indicated by horizontal axis of right panel.

doi:10.1371/journal.pcbi.1004576.g005

when inhibitory modulation is near 1. Such power-laws are expected for continuous phase transitions.

Finally, our findings highlight a promising hypothesis for future research; changes in cortical state due to changes in behavioral context [1] may tune sensory dynamic range to suit the needs of the organism. For example, a highly focused task may benefit from a state with lower dynamic range, away from criticality. In contrast, a critical cortical state with high dynamic range may be optimal when vigilance or readiness for unknown input is important. Confirmation of this hypothesis would establish a general principle of sensory information processing: sensory dynamic range can be optimized by tuning the cortical state and maximized specifically in the critical cortical state.

Materials and Methods

Electrophysiology

All procedures were carried out in accordance with the recommendations in the Guide for the Care and Use of Laboratory Animals of the National Institutes of Health and approved by University of Arkansas Institutional Animal Care and Use Committee (protocol #12025). We studied adult male rats (n = 13, 328±54 g; *Rattus Norvegicus*, Sprague-Dawley outbred, Harlan Laboratories, TX, USA). Anesthesia was induced with isoflurane inhalation and maintained with urethane (1.5 g/kg body weight (bw) dissolved in saline, intraperitoneal injection (ip)). Dexamethasone (2 mg/kg bw, ip) and atropine sulphate (0.4 mg/kg bw, ip) were administered before performing a 2 mm x 2 mm craniotomy over barrel cortex (1 to 3 mm posterior from bregma, 5 to 7 mm lateral from midline).

Extracellular voltage was recorded using 32-channel microelectrode arrays. The electrode arrays were comprised of 8 shanks with 4 electrodes per shank, 200 μm inter-electrode distance, 400 μm inter-shank distance (A468-5 mm-200–400-177-A32, NeuroNexus, MI, USA). Each shank was made of silicon and each electrode contact was made of iridium. Shanks were 50 μm x 15 μm in cross section. Electrode impedances were approximately 1 MΩ at 1 kHz. Insertion depth was 650 μm, centered 2 mm posterior from bregma and 6 mm lateral from midline. Voltages were measured with respect to an AgCl ground pellet placed in the saline-soaked gel foams, which protect the exposed tissue surrounding the insertion site. Voltages were digitized with 30 kHz sample rate (Cereplex + Cerebus, Blackrock Microsystems, UT, USA). Recordings were filtered between 300 and 3000 Hz and thresholded at -3 SD to detect multi-unit activity (MUA).

Multi-whisker stimulation and detection

All whiskers were trimmed except 2–4 whiskers from rows A-C and arcs 1–4. A computer-automated, pressure-controlled air puff (1 s duration) was used to deliver 10 different puff intensities, each repeated 20 times in pseudorandom order at 7 s intervals. As shown in Fig 1 and previously described [35], two-dimensional (rostrocaudal and mediolateral) multi-whisker motion was measured with millisecond, micron precision using two line cameras (LC100, Thorlabs Inc, NJ, USA). Response curves were based on the speed of the whisker which evoked the largest MUA neural response, which we call the dominant whisker.

Pharmacology

Up to nine 20 min recordings were conducted with each rat. First, three recordings were performed with no direct manipulation of inhibition (n = 32, indirect effects may be imposed by anesthetics [36] and atropine sulfate). Then, three recordings were performed with a drug topically applied via gel foam pieces soaked in saline mixed with drug. Finally, three wash experiments were performed with drug-free gel foams. Three drug conditions were studied (one condition per rat): 1) 20 μM muscimol (6 rats, 15 recordings), 2) 20 μM bicuculline methiodide (4 rats, 10 experiments), 3) 40 μM bicuculline methiodide (3 rats, 8 experiments). The wash condition for bicuculline typically returned to activity similar to that measured in pre-drug experiments, but this was not the case for muscimol.

Computational model

The model was comprised of N = 1000 binary, probabilistic, integrate-and-fire neurons. At each time t, the state $s_i(t)$ of neuron i was 0 (quiescent) or 1 (firing). At each time there was a probability p_{ext} that each neuron would fire due to external input and a probability $p_i(t)$ that it would fire due to input from other neurons $I_i(t)$,

$$p_i(t) = \begin{cases} 1 & for\ I_i(t) > 1 \\ I_i(t) & for\ 0 \leq I_i(t) \leq 1 \\ 0 & for\ I_i(t) < 0 \end{cases},$$

$$I_i(t) = \left(\sum_{j \neq i} W_{ij} s_j(t-1) \right) \frac{1}{h_i(t)} ,$$

where $h_i(t)$ depends on recent firing history

$$h_i(t) = \sum_{\tau = t-T}^{t} s_i(\tau).$$

In cases where this sum was zero, we set h to 1. Note that T plays an important role determining the character of the ongoing network dynamics as well as the shape of the stimulus response curves presented in Results (see Fig 5). For the results shown in Figs 2, 3, 4 and 5C, we set T = 80 ms (assuming that one model time step takes 1 ms) to obtain good qualitative fit with observed experimental results. For the results shown in Fig 5B, we set T = 0, i.e. there was no history-dependent depression.

The default synaptic weight matrix W is constructed as follows. First, all entries are drawn from a uniform distribution [0, 1]. Second, 20% of neurons are designated as inhibitory and the corresponding columns of W are multiplied by -1. Third, all entries are multiplied by a constant to enforce that the largest eigenvalue of W is 1. This third step ensures that the network operates at criticality [21]. Thus, the network topology is all-to-all coupling, but with non-uniform coupling strength. To model pharmacological manipulation of inhibition we multiply all the negative entries of W by a constant ranging from 0 (model of strong GABA antagonist) to 3 (model of strong GABA agonist). This is the quantity labeled 'inhibitory modulation' in Figs 2, 3, 4 and 5. These manipulations change the largest eigenvalue of W, thus pushing the system away from criticality.

To simulate the onset of sensory stimulation, p_{ext} undergoes a step increase. The pre-stimulus low rate ($p_{ext} = 5 \times 10^{-6}$) resulted in 5 externally-driven spikes per second for the entire network, assuming that one model time step was 1 ms. The during-stimulus high rate was fixed

during a single trial, but varied across trials to model different intensities of sensory stimulation. High rates ranged from $p_{ext} = 5 \times 10^{-6}$ to $p_{ext} = 1 \times 10^{-3}$, i.e. generating 5 to 1000 externally-driven spikes per second for the entire network. Each level of p_{ext} we repeated 20 times, just as each experimental stimulus intensity was repeated 20 times. We note that increasing p_{ext} to values approaching 1 does result in a saturation of network activity, which changes the shape of the stimulus-response curves and changes dynamic range. However, such highly saturated response-curves were not observed in our experiments. The chosen range of p_{ext}, better fits our experimental observations.

Experimental data analysis

MUA spike count time series were based on time bins of duration DT = 7.5±3 ms (mean±SD), depending on the number of good electrodes, signal/noise, and baseline spike rates for each animal. The threshold for avalanche detection was TH = 11±5 spikes per time bin. For experiments with overall higher MUA spike rates, presumably due to differences in experimental details like electrode quality, we chose smaller DT and smaller TH. However, this experiment-specific tuning was not necessary to support our conclusions. Indeed, we found that changes in the choice of time bin durations (in the range 5 ms > DT > 20 ms) and avalanche thresholds (in the range 5 > TH > 20) can cause small changes in the shape of the avalanche distribution (S1 Fig) and, consequently, small changes in κ. However, we emphasize that our primary conclusion–peak dynamic range near κ = 1 –was robust to such parameter variations (S2 Fig). Deviation from the reference power-law (-1.5 exponent) was quantified with κ, which is a previously developed non-parametric measure with similarities to a Kolmogorov-Smirnov statistic [19]; κ equals 1 plus the sum of 10 differences (logarithmically spaced) between the observed avalanche size distribution (recast as a cumulative distribution) and a perfect power-law (in cumulative form). In the summary plots of κ versus correlation and κ versus dynamic range, experiments were grouped according to their κ values into 13 equally spaced κ bins.

For dynamic range calculations, each point in the response curve was the average MUA response for a range of whisker speeds. The binning of whisker speeds was based on 10 equally spaced values spanning the range of observed speeds. Finally, the response curve was fit with a sigmoid function,

$$f(x) = \frac{R_{max}}{1 + e^{-b(x-c)}} + R_{min}$$

where R_{min} was defined as the ongoing spike count per time bin and the constants R_{max}, b, and c were fitting parameters. This fit function was used to compute dynamic range, as defined in previous studies [19,20]. First, two stimulus levels, S_{10} and S_{90} are defined as those stimuli which give rise to response R_{10} and R_{90} as illustrated in Fig 4D (inset). R_{10} is defined as the response level R_{min}+0.1(R_{max}- R_{min}) and R_{90} is defined as the response level R_{min}+0.9(R_{max}-R_{min}). Finally, we define dynamic range as

$$\Delta = 10 \log_{10} \frac{S_{90}}{S_{10}}$$

Model data analysis

Analysis of model data paralleled the experimental data analysis with a few exceptions. MUA spike count time series were based on time bins of duration 1 time step. The threshold for avalanche detection was 10 spikes per time bin. For computing stimulus-response curves, the

response was defined as the total number of spikes from the entire network during the first 200 time steps following onset of stimulation (increase in p_{ext}). For dynamic range calculations based on model data, we did not fit the response curves with a sigmoid because they were less noisy than the experimental response curves. The correlation coefficients in Fig 3 were computed in a way to mimic the experiments, as follows. First, the 1000 model neurons were broken up into 32 groups, like the 32 electrodes in experiments. Then, a spike count timeseries was created for each group. Finally, all pairwise zero-lag Pearson correlation coefficients were computed and averaged together.

Supporting Information

S1 Fig. Continuum of cortical states is robust to variations in the analysis parameters TH and DT. Shown are all of the avalanche size distributions (with log-log axes), vertically shifted and color coded according to their κ values (high and red for high κ; low and blue for low κ). A subset of these distributions is shown in Fig 2D of the main manuscript. Each panel shows distributions created with different parameters TH and DT. We tested this for TH = 5, 10, and 20 spikes (left to right) and DT = 5, 10, and 20 ms (top to bottom). Robust to these parameter changes is the general observation of a continuum of cortical states ranging from weakly correlated small avalanches (blue) to strongly correlated large avalanches (red). As DT increases (top to bottom), κ systematically tends toward higher values. Changing TH (left to right) also can change κ, but without a clear systematic trend. The parameter DT defines the duration of the time bins used to make the spike count time series. The parameter TH is the threshold for defining avalanches. (See Materials and Methods for more description of these parameters.)
(PDF)

S2 Fig. Peak dynamic range near κ = 1 is robust to variations in the analysis parameters TH and DT. Shown are summary plots of dynamic range Δ versus deviation from power-law κ. These are comparable to the data shown in Fig 4D of the main manuscript. Since κ depends on the analysis parameters DT and TH, as shown in S1 Fig, the Δ versus κ relationship could also depend on these parameters. We tested this for TH = 5, 10, and 20 spikes (left to right) and DT = 5, 10, and 20 ms (top to bottom). The central panel is closest to the parameter values used for the analysis shown in Fig 4D. Most of these parameter combinations show a clear peak in dynamic range near κ = 1. Thus, we conclude that our primary finding is largely robust to these changes in analysis parameters.
(PDF)

Author Contributions

Conceived and designed the experiments: WLS SHG KM TTH SKG. Performed the experiments: WLS SHG KM TTH KLM. Analyzed the data: WLS TTH. Wrote the paper: WLS SHG.

References

1. Harris KD, Thiele A (2011) Cortical state and attention. Nat Rev Neurosci 12: 509–523. doi: 10.1038/nrn3084 PMID: 21829219
2. Renart A, Machens CK (2014) Variability in neural activity and behavior. Curr Opin Neurobiol 25C: 211–220. doi: 10.1016/j.conb.2014.02.013
3. Castro-Alamancos MA (2009) Cortical up and activated states: implications for sensory information processing. Neuroscientist 15: 625–634. doi: 10.1177/1073858409333074 PMID: 19321459
4. Steriade M (2003) Neuronal Substrates of Sleep and Epilepsy. Cambridge: Cambridge University Press. doi: 10.1017/CBO9780511541711

5. Castro-Alamancos MA (2004) Absence of rapid sensory adaptation in neocortex during information processing states. Neuron 41: 455–464. PMID: 14766183
6. Poulet JF a, Fernandez LMJ, Crochet S, Petersen CCH (2012) Thalamic control of cortical states. Nat Neurosci 15: 370–372. doi: 10.1038/nn.3035 PMID: 22267163
7. Goard M, Dan Y (2009) Basal forebrain activation enhances cortical coding of natural scenes. Nat Neurosci 12: 1444–1449. doi: 10.1038/nn.2402 PMID: 19801988
8. Castro-Alamancos MA, Gulati T (2014) Neuromodulators Produce Distinct Activated States in Neocortex. J Neurosci 34: 12353–12367. doi: 10.1523/JNEUROSCI.1858-14.2014 PMID: 25209276
9. Hirata A, Castro-Alamancos MA (2011) Effects of cortical activation on sensory responses in barrel cortex. J Neurophysiol 105: 1495–1505. doi: 10.1152/jn.01085.2010 PMID: 21273311
10. Supp GGG, Siegel M, Hipp JFF, Engel AKK (2011) Cortical hypersynchrony predicts breakdown of sensory processing during loss of consciousness. Curr Biol 21: 1988–1993. doi: 10.1016/j.cub.2011.10.017 PMID: 22100063
11. Scott G, Fagerholm ED, Mutoh H, Leech R, Sharp DJ, et al. (2014) Voltage Imaging of Waking Mouse Cortex Reveals Emergence of Critical Neuronal Dynamics. J Neurosci 34: 16611–16620. doi: 10.1523/JNEUROSCI.3474-14.2014 PMID: 25505314
12. Pachitariu XM, Lyamzin DR, Sahani M, Lesica NA (2015) State-Dependent Population Coding in Primary Auditory Cortex. 35: 2058–2073. doi: 10.1523/JNEUROSCI.3318-14.2015 PMID: 25653363
13. Shew WL, Yang H, Yu S, Roy R, Plenz D (2011) Information Capacity and Transmission Are Maximized in Balanced Cortical Networks with Neuronal Avalanches. J Neurosci 31: 55–63. doi: 10.1523/JNEUROSCI.4637-10.2011 PMID: 21209189
14. Bermudez Contreras EJ, Schjetnan AGP, Muhammad A, Bartho P, McNaughton BL, et al. (2013) Formation and Reverberation of Sequential Neural Activity Patterns Evoked by Sensory Stimulation Are Enhanced during Cortical Desynchronization. Neuron 79: 555–566. doi: 10.1016/j.neuron.2013.06.013 PMID: 23932001
15. Scholvinck ML, Saleem a. B, Benucci A, Harris KD, Carandini M (2015) Cortical State Determines Global Variability and Correlations in Visual Cortex. J Neurosci 35: 170–178. doi: 10.1523/JNEUROSCI.4994-13.2015 PMID: 25568112
16. Petersen CCH, Hahn TTG, Mehta M, Grinvald A, Sakmann B (2003) Interaction of sensory responses with spontaneous depolarization in layer 2/3 barrel cortex. Proc Natl Acad Sci U S A 100: 13638–13643. doi: 10.1073/pnas.2235811100 PMID: 14595013
17. Marguet SL, Harris KD (2011) State-dependent representation of amplitude-modulated noise stimuli in rat auditory cortex. J Neurosci 31: 6414–6420. doi: 10.1523/JNEUROSCI.5773-10.2011 PMID: 21525282
18. Reimer J, Froudarakis E, Cadwell CR, Yatsenko D, Denfield GH, et al. (2014) Pupil Fluctuations Track Fast Switching of Cortical States during Quiet Wakefulness. Neuron 84: 355–362. doi: 10.1016/j.neuron.2014.09.033 PMID: 25374359
19. Shew WL, Yang H, Petermann T, Roy R, Plenz D (2009) Neuronal Avalanches Imply Maximum Dynamic Range in Cortical Networks at Criticality. J Neurosci 29: 15595–15600. doi: 10.1523/JNEUROSCI.3864-09.2009 PMID: 20007483
20. Kinouchi O, Copelli M (2006) Optimal dynamical range of excitable networks at criticality. Nat Phys 2: 348–351. doi: 10.1038/nphys289
21. Larremore DB, Shew WL, Restrepo JG (2011) Predicting Criticality and Dynamic Range in Complex Networks: Effects of Topology. Phys Rev Lett 106: 1–4. doi: 10.1103/PhysRevLett.106.058101
22. Pei S, Tang S, Yan S, Jiang S, Zhang X, et al. (2012) How to enhance the dynamic range of excitatory-inhibitory excitable networks. Phys Rev E 86: 1–7. doi: 10.1103/PhysRevE.86.021909
23. Plenz D, Niebur E, Schuster HG (2014) Criticality in Neural Systems. Plenz D, Niebur E, Schuster HG, editors Weinheim, Germany: Wiley.
24. Shew WL, Plenz D (2013) The functional benefits of criticality in the cortex. Neuroscientist 19: 88–100. doi: 10.1177/1073858412445487 PMID: 22627091
25. Beggs JM, Plenz D (2003) Neuronal avalanches in neocortical circuits. J Neurosci 23: 11167–11177. PMID: 14657176
26. Larremore DB, Shew WL, Ott E, Sorrentino F, Restrepo JG (2014) Inhibition Causes Ceaseless Dynamics in Networks of Excitable Nodes. Phys Rev Lett 112: 138103. doi: 10.1103/PhysRevLett.112.138103 PMID: 24745460
27. Poil S- S, Hardstone R, Mansvelder HD, Linkenkaer-Hansen K (2012) Critical-state dynamics of avalanches and oscillations jointly emerge from balanced excitation/inhibition in neuronal networks. J Neurosci 32: 9817–9823. doi: 10.1523/JNEUROSCI.5990-11.2012 PMID: 22815496

28. Buzsáki G, Mizuseki K (2014) The log-dynamic brain: how skewed distributions affect network operations. Nat Rev Neurosci. doi: 10.1038/nrn3687 PMID: 24569488
29. Muñoz M, Dickman R, Vespignani A, Zapperi S (1999) Avalanche and spreading exponents in systems with absorbing states. Phys Rev E 59: 6175–6179. doi: 10.1103/PhysRevE.59.6175
30. Larremore DB, Carpenter MY, Ott E, Restrepo JG (2012) Statistical properties of avalanches in networks. Phys Rev E 85: 066131. doi: 10.1103/PhysRevE.85.066131
31. Averbeck BB, Latham PE, Pouget A (2006) Neural correlations, population coding and computation. Nat Rev Neurosci 7: 358–366. doi: 10.1038/nrn1888 PMID: 16760916
32. Jacobs AL, Fridman G, Douglas RM, Alam NM, Latham PE, et al. (2009) Ruling out and ruling in neural codes. Proc Natl Acad Sci U S A 106: 5936–5941. doi: 10.1073/pnas.0900573106 PMID: 19297621
33. Panzeri S, Schultz SR, Treves a, Rolls ET (1999) Correlations and the encoding of information in the nervous system. Proc Biol Sci 266: 1001–1012. doi: 10.1098/rspb.1999.0736 PMID: 10610508
34. Buckley C, Nowotny T (2011) Multiscale Model of an Inhibitory Network Shows Optimal Properties near Bifurcation. Phys Rev Lett 106. doi: 10.1103/PhysRevLett.106.238109 PMID: 21770552
35. Grady SK, Hoang TT, Gautam SH, Shew WL (2013) Millisecond, Micron Precision Multi-Whisker Detector. PLoS One 8: e73357. doi: 10.1371/journal.pone.0073357 PMID: 24023861
36. Populin LC (2005) Anesthetics change the excitation/inhibition balance that governs sensory processing in the cat superior colliculus. J Neurosci 25: 5903–5914. doi: 10.1523/JNEUROSCI.1147-05.2005 PMID: 15976079
